# A Physiologically Based Pharmacokinetic Approach to Recommend an Individual Dose of Tacrolimus in Adult Heart Transplant Recipients

**DOI:** 10.3390/pharmaceutics15112580

**Published:** 2023-11-03

**Authors:** Ling Pei, Run Li, Hong Zhou, Wenxin Du, Yajie Gu, Yingshuo Jiang, Yongqing Wang, Xin Chen, Jianguo Sun, Junrong Zhu

**Affiliations:** 1 Department of Pharmacy, Nanjing First Hospital, China Pharmaceutical University, Nanjing 210006, China; 2 Department of Pharmacy, Nanjing First Hospital, Nanjing Hospital Affiliated to Nanjing Medical University, Nanjing 210006, China; 3 Key Laboratory of Drug Metabolism and Pharmacokinetics, China Pharmaceutical University, Nanjing 210009, China; 4 Department of Pharmacy, Union Hospital, Tongji Medical College, Huazhong University of Science and Technology, Wuhan 430022, China; 5 Department of Cardiothoracic Surgery, Nanjing First Hospital, Nanjing Hospital Affiliated to Nanjing Medical University, Nanjing 210006, China; 6 Research Division of Clinical Pharmacology, First Affiliated Hospital of Nanjing Medical University, Nanjing 210029, China

**Keywords:** physiologically based pharmacokinetics model, heart transplantation, tacrolimus, therapeutic drug monitoring, genetic polymorphism

## Abstract

Tacrolimus is the principal immunosuppressive drug which is administered after heart transplantation. Managing tacrolimus therapy is challenging due to a narrow therapeutic index and wide pharmacokinetic (PK) variability. We aimed to establish a physiologically based pharmacokinetic (PBPK) model of tacrolimus in adult heart transplant recipients to optimize dose regimens in clinical practice. A 15-compartment full-PBPK model (Simbiology^®^ Simulator, version 5.8.2) was developed using clinical observations from 115 heart transplant recipients. This study detected 20 genotypes associated with tacrolimus metabolism. CYP3A5*3 (rs776746), CYP3A4*18B (rs2242480), and IL-10 G-1082A (rs1800896) were identified as significant genetic covariates in tacrolimus pharmacokinetics. The PBPK model was evaluated using goodness-of-fit (GOF) and external evaluation. The predicted peak blood concentration (C_max_) and area under the drug concentration–time curve (AUC) were all within a two-fold value of the observations (fold error of 0.68–1.22 for C_max_ and 0.72–1.16 for AUC). The patients with the CYP3A5*3/*3 genotype had a 1.60-fold increase in predicted AUC compared to the patients with the CYP3A5*1 allele, and the ratio of the AUC with voriconazole to alone was 5.80 when using the PBPK model. Based on the simulation results, the tacrolimus dosing regimen after heart transplantation was optimized. This is the first PBPK model used to predict the PK of tacrolimus in adult heart transplant recipients, and it can serve as a starting point for research on immunosuppressive drug therapy in heart transplant patients.

## 1. Introduction

Tacrolimus is integral to immunosuppressive drug therapy for heart transplant recipients [1]. Dosing adjustment is recommended based on therapeutic drug monitoring (TDM) owing to its narrow therapeutic window, wide intra- and inter-individual pharmacokinetic (PK) variability, and nonlinear pharmacokinetics [2,3]. In clinical practice, oral tacrolimus usually reaches steady-state blood concentrations in approximately 72 h, and the whole blood trough concentration (C_trough_) level should be 10 to 15 ng/mL during the early postoperative period (days 0–60) [4,5]. The pharmacokinetics of tacrolimus are complex and highly variable, especially after oral administration. The required dose to achieve similar exposures can vary between patients by over a 10-fold difference [6]. The optimal dosing strategy for rapidly achieving therapeutic concentrations remains problematic in early postoperative unfavorable clinical conditions, despite reactive adjustments to frequent tacrolimus TDM.

Nonlinear pharmacokinetics are related to drug properties and the saturation of hepatic metabolism. Tacrolimus, for instance, exhibits concentration-dependent blood binding and is primarily distributed in erythrocytes (85–95%). Individual differences in hematocrit lead to variability [3,7]. Tacrolimus is primarily metabolized in the liver by CYP3A4 and CYP3A5, making it susceptible to drug interactions [8]. Individuals with at least one CYP3A5*1 allele express the CYP3A5 protein at high levels, which is referred to as the extensive metabolite. In contrast, individuals with CYP3A5*3/*3 variants express little or no CYP3A5 protein, indicating poor metabolism [8]. CYP3A5 single nucleotide polymorphisms (SNPs) are estimated to account for 40–50% of the interindividual pharmacokinetic variation of tacrolimus [9]. Clinical Pharmacogenetics Implementation Consortium (CPIC) guidelines recommend adjusting the dose of medication based on the CYP3A5 genotype [10]. P-glycoprotein (P-gp) is also an important determinant of tacrolimus PK [11]. In addition, the upstream regulatory gene POR of CYP3A [12] and the immune genes IL-6 and IL-10 have also been reported to be related to the pharmacokinetics of tacrolimus. Still, the relevant studies could be more extensive and their results need further verification [13,14]. Therefore, the 20 metabolic genes of tacrolimus were detected.

The physiologically based pharmacokinetic model (PBPK) has been recommended to predict the impact of physiological, physiochemical, pharmacogenetic factors, and concomitant medication on drug exposure [15,16]. At present, most studies have focused on the pharmacokinetics of tacrolimus in liver transplantation [17,18]. The top-down approach to pharmacokinetic studies, based on clinical laboratory data in heart transplant patients, limits the potential for individualized treatment [19,20]. Compared to other transplant groups, heart transplant recipients require frequent dose adjustments of tacrolimus due to continuous changes in tacrolimus metabolism and binding capacity, and increased co-administered medication, such as voriconazole. Additionally, low cardiac output syndrome and acute gastric mucosal injury contribute to oral malabsorption, leading to a greater variability in drug exposure [2,21].

Therefore, a PBPK model for tacrolimus was constructed to determine the sources of influence on tacrolimus PK variation and predict PK among adult heart transplant patients. The approach combined bottom-up analysis with top-down population pharmacokinetic (PopPK) analysis [18,22]. Determining these relevant covariates helps clinicians accurately estimate the individualized initial dose of tacrolimus in the early post-transplant period.

## 2. Materials and Methods

### 2.1. Patient Selection

The patients who received heart transplantation at Nanjing First Hospital from November 2012 to January 2023 were retrospectively included. The inclusion criteria were as follows: adult heart transplant patients (≥18 years); immunosuppressant therapy based on tacrolimus (Prograf^®^, Astellas, Dublin, Ireland), with mycophenolate and corticosteroids. The exclusion criteria were as follows: multi-organ transplantation; incomplete clinical data. A total of 115 patients were included for analysis.

### 2.2. Data Collection

Clinical data were collected during post-transplantation (from day 1 to day 30) according to electronic medical records, including (1) tacrolimus dosing information; (2) tacrolimus steady-state trough concentrations (C_trough_); (3) demographic characteristics: weight, height, age, gender, postoperative day (POD); (4) concomitant CYP3A inhibition medications: voriconazole, fluconazole, calcium antagonists, proton pump inhibitors, and amiodarone; (5) biochemical parameters: red blood cell count (RBC), white blood cell count (WBC), hematocrit (HCT), hemoglobin (HGB), alanine transaminase (ALT), aspartate aminotransferase (AST), alkaline phosphatase (ALP), albumin concentration (ALB), phosphocreatine kinase (CK), lactate dehydrogenase (LDH), activated partial thromboplastin time (APTT), prothrombin time (PT), thrombin time (TT), PT-INR, total bilirubin (TBIL), blood uric nitrogen (BUN), serum creatinine (SCR), and glucose. This study was approved by the Nanjing Medical University College Ethics Committee (ethical code: KY20190404-03-KS-01).

### 2.3. Sample Collection and Analysis

Heart tissue samples (1 mg) and whole blood (2 mL) samples were obtained from heart transplant recipients in EDTA anticoagulated sterile tubes. Samples were extracted using the Magbeads Blood DNA Kit (Cowin Biotech Co., Ltd., Taizhou, China) and FastPure Cell/Tissue DNA Isolation Mini Kit (Vazyme Biotech Co., Ltd., Nanjing, China). Single nucleotide polymorphisms (SNPs) were determined with Equalbit 1 × dsDNA HS Assay Kit (Vazyme Biotech Co., Ltd., Nanjing, China). Whole blood tacrolimus concentrations were determined usnig CMIA. The lower limit of quantification of the assay was 2 ng/mL, and the quantitative range was 2–30 ng/mL.

### 2.4. Population PK Analysis

Population PK analysis was conducted with the Phoenix NLME 8.3 program (Certara, St Louis, MI, USA). The covariate model consisting of a one-compartment model with first-order absorption and elimination was constructed to determine covariates using a stepwise forward inclusion (*p* < 0.05) backward exclusion (*p* < 0.01) regression approach [23]. It was evaluated using a prediction-corrected visual predictive check (pcVPC) and the bootstrap approach. The process of the population PK model development is provided in the Supplementary Materials.

### 2.5. Model Development

The full-PBPK model was developed using Simbiology^®^ (v.5.8.2, MathWorks, Natick, MA, USA). A perfusion-limited model consisted of 15 compartments representing important tissues and organs [24,25]. The schematic diagram of the PBPK model is presented in Figure S1. It is assumed that all organs were well-stirred and drug distribution is limited by blood flow [26]. Drug parameters and human anatomical and physiological parameters are listed in Table 1 and Table S1, respectively.

The concentration dynamics in the tissue compartment are described as follows [27]:

(1) $$\begin{array}{c} \;V_{T}\frac{dC_{T}}{\mathrm{dt}}={\;Q}_{T}\times {(C}_{A}-\frac{C_{T}}{\frac{K_{T:p}}{\mathrm{BPR}}}) \end{array}$$

where V = volume (L), C = blood concentration (ng/mL), Q = blood flow (L/h), T = tissues, A = arteries, K_T:p_ = tissue-to-plasma partition coefficient, BPR = blood-to-plasma concentration ratio. The volume of each organ, V, was adjusted in relation to the bodyweight (BW) and the proportion of adipose tissue [24].

Table S2 summarized the tissue-to-plasma partition coefficients estimated using Rodgers and Rowland’s method [28,29]. The average K_T:p_ of tacrolimus obtained using Rodgers and Rowland’s formulas was 1.3. The average K_T:p_ of mice reported in the literature was 11.9, which was nearly 10-fold higher [30]. Therefore, the human K_T:p_ was applied by a scaling factor a.

After administration, the concentration dynamics in the absorption compartment were described by Equation (2) as follows:

(2) $$\begin{array}{c} \;{\;V}_{T}\frac{dC_{T}}{\mathrm{dt}}={\;Q}_{T}\times {(C}_{A}-\frac{C_{T}}{\frac{K_{T:p}}{\mathrm{BPR}}})+F_{g}\times K_{a}\times Dose \end{array}$$

where F_g_ = the fraction of dose absorbed, k_a_ = the first-order absorption rate constant (1/h), Dose = the dose of tacrolimus. F_g_ was assumed to be constant (F_g_ = 0.2) [26].

The liver was considered the only elimination organ. The tissue extraction ratio was calculated using Equation (3) [31].

(3) $$\begin{array}{c} E=\frac{{\mathrm{fu}}_{b}\times {\mathrm{CL}}_{\mathrm{int}}}{Q_{\mathrm{liver}}+{\mathrm{fu}}_{b}\times {\mathrm{CL}}_{\mathrm{int}}} \end{array}$$

where E = the liver extraction ratio; CL_int_ = the intrinsic clearance (L/h); fu_b_ = the unbound fraction in the blood, and it was calculated using Equation (4).

(4) $${\mathrm{fu}}_{b}=\frac{{\mathrm{fu}}_{p}}{\mathrm{BPR}}$$

where fu_p_ = the unbound fraction in the plasma [32].

BPR was related to hematocrit [33]. It was calculated using Equation (5).

(5) $$\begin{array}{c} BPR=1+\frac{B_{\max}\times \mathrm{HCT}}{K_{D}\times {\mathrm{HCT}}_{m}} \end{array}$$

where B_max_ = the binding capacity, K_D_ = the affinity constant. HCT_m_ = the median hematocrit in the heart transplant population.

The formula contains three parts in the elimination part: CYP3A5, CYP3A4, and other paths:

(6) $$\begin{array}{c} {\mathrm{CL}}_{\mathrm{liver}}={[(\mathrm{fm}}_{\mathrm{CYP}3A5}\times {\mathrm{FA}}_{\mathrm{CYP}3A5}+{\mathrm{fm}}_{\mathrm{CYP}3A4}\times {\mathrm{FA}}_{\mathrm{CYP}3A4})+{\mathrm{fm}}_{\mathrm{other}}]\times Q_{\mathrm{liver}}\times E \end{array}$$

where fm_CYP3A5_ = the fraction of tacrolimus hepatic clearance by CYP3A5, fm_CYP3A4_ = the fraction of tacrolimus hepatic clearance using CYP3A4, FA_CYP3A5_ = the activity level with CYP3A5, FA_CYP3A4_ = the activity level of CYP3A4. The value of fm_CYP3A_ was calculated by oral clearance of CYP3A expression and non-expression [34]. The PBPK model combined a top-down approach and used a nonlinear regression method to fit the tacrolimus concentrations of healthy adults and 115 patients in a clinical study to estimate the key parameters (the first-order absorption rate constant (k_a_), the binding capacity (B_max_), the affinity constant (K_D_), the intrinsic clearance (CL_int_), the activity level of CYP3A4 (FA_CYP3A4_), the activity level of CYP3A5 (FA_CYP3A5_)).

### 2.6. Model Evaluation

Graphical evaluation was used for the performance of the tacrolimus PBPK model: (1) visual inspections between the observed data and the 90% confidence interval of the simulated blood concentration–time profiles were carried out; (2) a goodness-of-fit plot (R^2^) was generated from a predicted and observed concentration [35]. In addition, the predicted area under the concentration–time curve (AUC) and peak blood concentration (C_max_) were compared with the corresponding observation. A two-fold error margin (0.5–2) was set as the inspection standard. The fold error was calculated using Equation (7) [36].

(7) $$\mathrm{Fold}\mathrm{error}\;=\frac{\mathrm{Predicted}\mathrm{value}}{\;\mathrm{Observed}\mathrm{value}}$$

The model was evaluated using the external dataset from heart transplant recipients at Wuhan Union Hospital. The key parameters were re-estimated for the PBPK model. Subsequently, voriconazole was used as a CYP3A inhibitor, and the parameters were integrated into the PBPK model to evaluate the interactions between voriconazole and tacrolimus in heart transplant patients. The drug–drug interaction (DDI) model development and evaluation are provided in the Supplementary Materials.

### 2.7. Evaluation of the Impact of Potential Covariates

To quantitatively evaluate the impact of important parameters on the changes in the pharmacokinetics of tacrolimus, the established PBPK model was used to simulate the oral administration of 2.5 mg tacrolimus to the CYP3A PMs population for sensitivity analysis. Mainly, the effects of drug-specific parameters and physiological parameters in PK parameters (AUC_0-last_, C_max_ and C_trough_) were evaluated. The range was defined for each parameter based on the initial (base) value, which varied within ±50%. The parameter was considered insensitive if the PK parameter varied by less than 10%.

### 2.8. Dosage Recommendations

Patients were categorized into various subgroups based on significant covariates. According to the PBPK model, 1000 simulations of PK profiles were conducted to determine the most suitable personalized dosing regimen for each subgroup. The simulations aimed to ensure that more than 90% of trough concentrations fell within the target concentration range (10–15 ng/mL), and the dosage regimen was generated based on the simulation results.

### 2.9. Statistical Methods

Statistical analyses were conducted using R (version 3.6.1, R Foundation for Statistical Computing, Vienna, Austria). The Shapiro–Wilk test was employed to analyze distribution characteristics. Measurement data with normal distributions were expressed as means ± standard deviations (x ± s). Non-normal distribution measurement data were expressed as medians (M) and interquartile ranges (IQR). Genotype analysis was conducted using the Hardy–Weinberg law, and the observed differences were statistically significant (*p* < 0.05).

## 3. Results

### 3.1. Population PK Study

The data for the population PK study were obtained from 443 tacrolimus plasma concentrations in 115 heart transplant recipients. Individuals in the model building dataset were primarily men (*n* = 93) and women (*n* = 22), with a median (range) age of 52.00 (46.00–61.00) years and a weight of 67.50 (57.50–75.00) kg, respectively. The dosing regimen of tacrolimus was 5.00 (4.00–6.00) mg/day. A total of 20 SNPs were detected in the samples of 86 transplant patients in this study. The results of the Hardy–Weinberg equilibrium law analysis showed that rs35599367, rs1135840, rs150461093, rs2229109, and rs4253728 had a *p*-value less than 0.05, which did not conform to the Hardy–Weinberg law of genetic equilibrium and was not included in the analysis. The demographics, laboratory data of the 115 heart transplant recipients, and the main genetic information are shown in Table 2. The remaining genetic information is summarized in Table S3.

The population estimates for the absorption rate constant (k_a_), apparent distribution volume (V_d_/F), and apparent clearance (CL/F) were 0.30 1/h, 656.80 L, and 12.35 L/h, respectively. The apparent clearance (CL/F) of tacrolimus showed a negative correlation with TBIL, voriconazole, CYP3A5*3 (rs776746), and IL-10 G-1082A (rs1800896). No relevant influencing variables were found for V_d_/F. The pcVPC of the final model is shown in Figure S2. The 5th, 50th, and 95th percentiles of the measured concentrations were almost overlaid with the 90% confidence intervals of the corresponding prediction percentiles for the final model. Furthermore, the bootstrap analysis, which was repeated 1000 times, was used to evaluate the stability of the final model. The parameter estimates of the final model were close to the bootstrap median estimates, and fell within the 2.5–97.5th percentage of the bootstrap parameter estimates, indicating that the parameter estimation was stable and reliable (Table S4).

**Figure 1:**
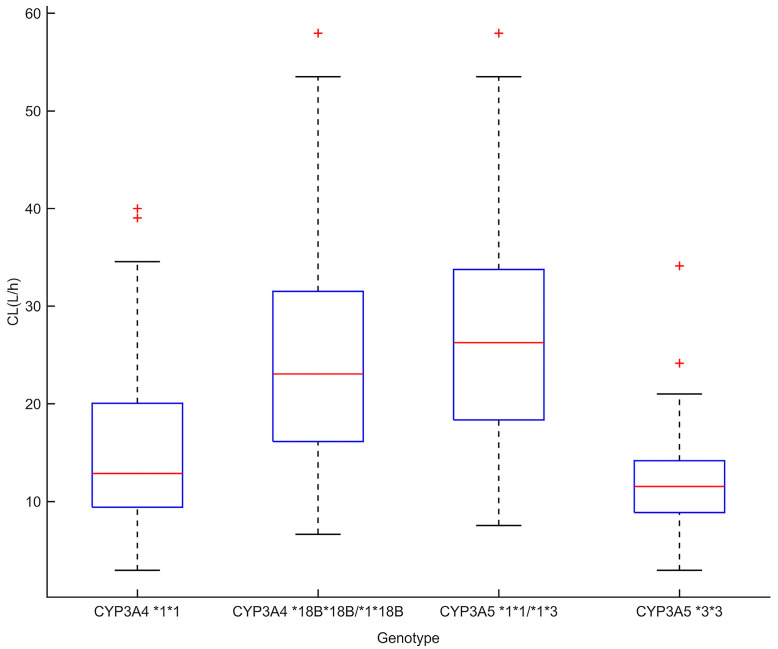
Tacrolimus clearance of CYP3A5*3 and CYP3A4*18B genotypes in heart transplant patients (*p* < 0.05). The box represents the Bayesian estimate of tacrolimus clearance in the population model. The edges of the box represent the 25th and 75th percentiles; the red lines inside the box represent the median; the dotted lines represent the 2.5th and 97.5th percentiles; the plus signs show outliers.

CYP3A4*18B genotypes were also significant covariates of CL/F. For CYP3A4*18B, there was a significant difference in CL/F of different genotypes: 25.4 ± 12.0 L/h for CYP3A4*18B*18B/*1*18B, and 16.3 ± 10.5 L/h for CYP3A4*1*1 (*p* < 0.001). For CYP3A5*3, the apparent clearance was 12.2 ± 6.0 L/h for CYP3A5*3*3, while it was 27.3 ± 11.5 L/h for individuals with at least one CYP3A5*1 allele (*p* < 0.001). The results are shown in Figure 1.

### 3.2. Model Development

**Figure 2:**
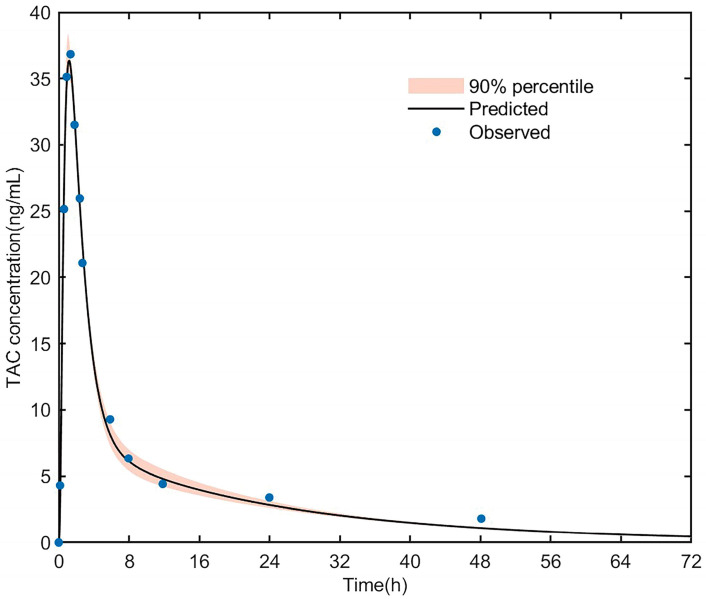
Simulated blood concentration–time profiles after a 5 mg oral dose of tacrolimus in healthy CYP3A5 expressers. The thick line represents the mean simulated concentration; the orange shadow represents the 5th and 95th percentiles of simulations; the solid dots represent the mean observed data. TAC, tacrolimus.

**Figure 3:**
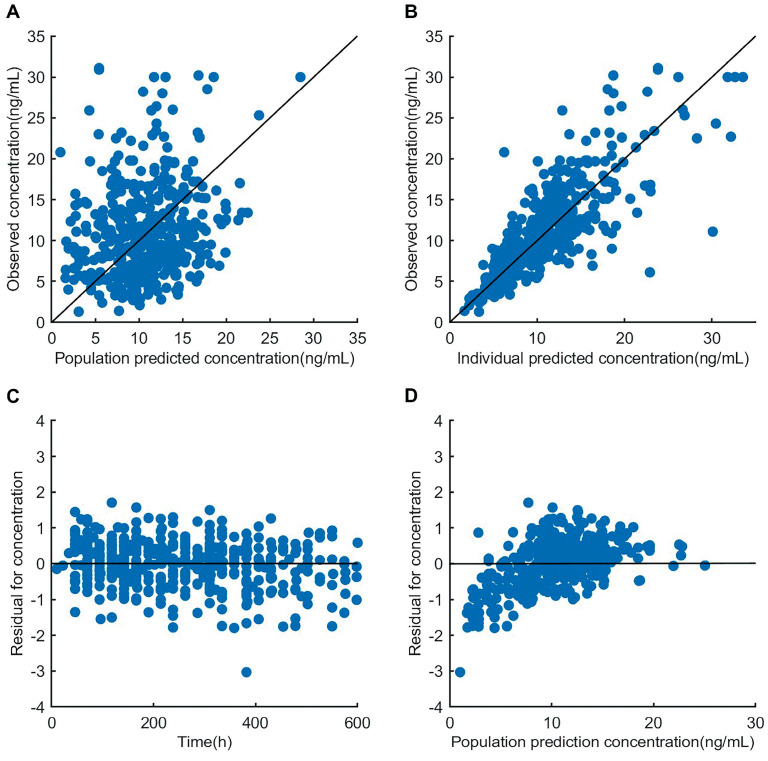
Goodness-of-fit plots of the PBPK model. (**A**) The plot of the observations versus population prediction. (**B**) The plot of the observations versus individual prediction. (**C**) The plot of residuals versus time. (**D**) The plot of residuals versus population prediction.

The PK data from healthy adults who received a single oral dose of tacrolimus (5 mg) were used for building the PBPK model of tacrolimus in healthy adults, as shown in Figure 2 [37]. The clinical data were used to develop the PBPK model of tacrolimus in heart transplant patients, and the goodness-of-fit plot is shown in Figure 3. Most of the residuals were within a range from −2 to 2 and showed no apparent trends with time, suggesting that the final model described the trend of concentration well.

To optimize parameters, the exponential error model with the minimum Akaike information criterion (AIC) and Bayesian information criterion (BIC) was selected as an error model. The estimated values of k_a_, B_max_, K_D_, and CL_int_ were 1.9 1/h, 145.9 μg/L, 7.2 μg/L, and 11,535 L/h. The scaling factor a was fitted to be 350. The parameters of healthy volunteers and heart transplant patients are shown in Table 3. After PK analysis, we identified those carrying CYP3A5*1 and CYP3A4*18B with extensive metabolizers (EM), and those carrying CYP3A5*3/*3 and CYP3A4*1/*1 with poor metabolizers (PM). The values of EM were set at 1 for FA_CYP3A5_ and FA_CYP3A4_. The values of PM were set at 0.3 and 0.5 for FA_CYP3A5_ and FA_CYP3A4_, respectively.

### 3.3. Model Evaluation

#### 3.3.1. PK Simulation of Tacrolimus in Healthy Adult

**Figure 4:**
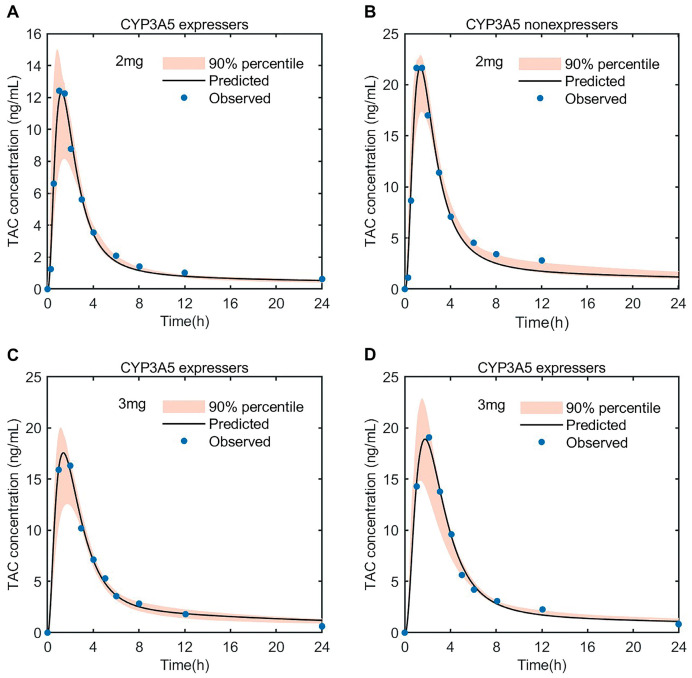
Predicted blood concentration–time profiles of tacrolimus after oral dose of 2 mg (**A**,**B**) and 3 mg (**C**,**D**) in healthy CYP3A5 expressers and non-expressers. The thick line represents the mean predicted concentration; the orange shadow represents the 5th and 95th percentiles of the prediction; the solid dots represent the mean observed data. TAC, tacrolimus.

As shown in Figure 4, the predicted concentration after the oral administration of different doses of tacrolimus (2, 3 mg) to subjects with a CYP3A5 expression and non-expression fit well with the observed drug data [38,39]. The results are shown in Figure S3, displaying linear regression with an R^2^ value = 0.9886. The pharmacokinetics (AUC, C_max_) are listed in Table S5; the fold error was in the range of 0.85–1.03. The predicted AUC of CYP3A5 non-expressers was nearly 2-fold higher than that of the CYP3A5 expressers, which also indicated that the CYP3A5 polymorphisms could significantly affect the pharmacokinetics of tacrolimus.

#### 3.3.2. PK Simulation of Tacrolimus in Adult Heart Transplant Patients

**Figure 5:**
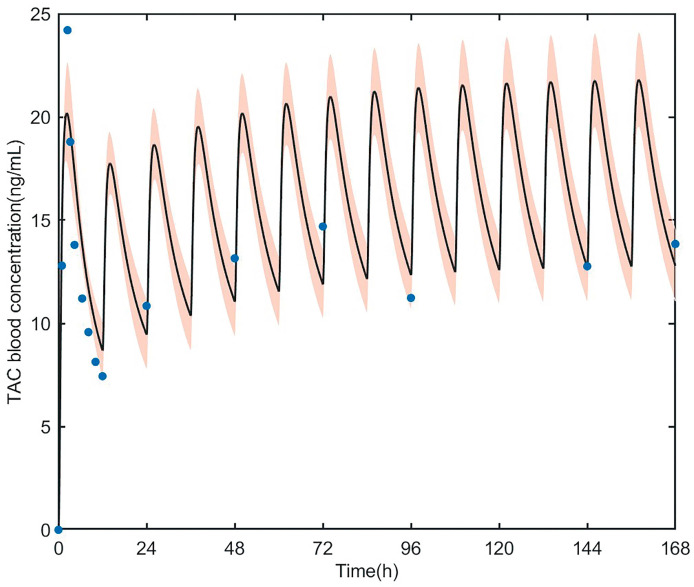
Predicted blood concentration–time profiles of tacrolimus in heart transplant patients. The thick line represents the mean predicted data; the orange shadow represents the 5th and 95th percentiles of the prediction; the blue solid dots represent observed data from heart transplant patients. TAC, tacrolimus.

**Figure 6:**
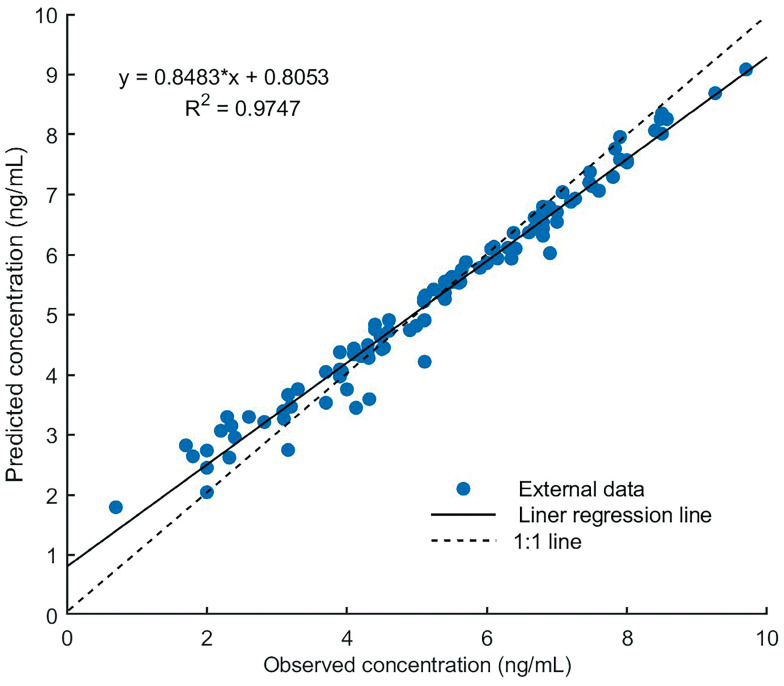
Performance of the tacrolimus PBPK model. Linear analysis for external validation of the predicted and observed tacrolimus concentration. “*” represents “×”.

The PBPK model accurately predicted tacrolimus PK in adult heart transplant recipients. The parameters estimated based on the data of Wuhan Union Hospital are shown in Table 3; there was no statistical difference compared with the model building data. First, as shown in Figure 5, the reliability of the pharmacokinetic model was verified by using tacrolimus blood concentration in a heart transplant patient (first dose = 0.057 mg/kg, followed by 0.028 mg/kg, q12h) [40], the observed concentrations were almost within 90% of the predicted concentration. The R^2^ value of external validation was 0.9747 (Figure 6), indicating the accurate performance of the final model. All predicted AUC_0–12h_ and C_max_ values fell within the 2-fold acceptance criteria that were indicated. The fold error of AUC_0–12h_ and C_max_ ranged from 0.68 to 1.22. All values can be found in Table 4.

Table S6 summarizes the parameters of voriconazole in the PBPK model. As shown in Figure S4, the predicted voriconazole concentration fits well with the observed concentration in healthy adults. As shown in Figure S5, PK datasets from three heart transplant patients (CYP3A5*1 expression) who were administered with voriconazole (the mean dose of tacrolimus = 1 mg, q12h; the dose of voriconazole = 200 mg, q12h, day 4–6) were utilized for DDI model development. In the DDI model verification, the linear analysis of the tacrolimus concentration combined with voriconazole is shown in Figure S6. The R^2^ value of validation was 0.9175, indicating the accurate performance of the model.

### 3.4. Impact of Covariates on Tacrolimus PKs

The impact of each parameter on tacrolimus pharmacokinetics was quantitatively assessed through local sensitivity analysis in patients with a poor metabolizer (PM). The simulated AUC_0-last_, C_max_, and C_trough_ of tacrolimus were more sensitive to fraction unbound in plasma (Table 5). Furthermore, the results showed that C_trough_ was more sensitive to several parameters.

**Figure 7:**
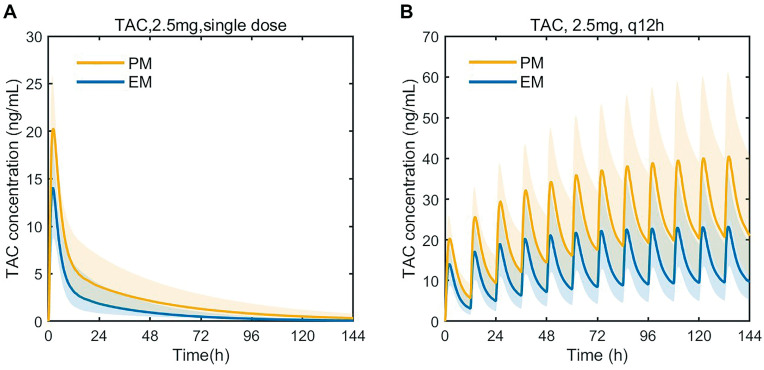
Predicted blood concentration–time profiles of tacrolimus after a single oral dose of 2.5 mg tacrolimus (**A**), predicted blood concentration–time profiles of tacrolimus after multidose of tacrolimus (2.5 mg, q12h for 6 days) (**B**). TAC, tacrolimus; EM: extensive metabolizers (CYP3A5*1*1 or *1*3 and CYP3A4*18B*18B or CYP3A4*1*18B); PM: poor metabolizers (CYP3A5*3*3 and CYP3A4*1*1). The thick line represents the mean predicted data; the yellow and blue shadow represents the 5th and 95th percentiles of the prediction.

PK prediction was performed according to the PBPK. In the single-dose simulation, the predicted AUC in PM was 191.91 ng/mL·h, which is a 1.60-fold increase in comparison to EM. In the multidose setting, the predicted C_trough_ of tacrolimus in PM was 21.00 ng/mL, which is a 2.18-fold increase in comparison to EM. The simulation is demonstrated in Figure 7. All values can be found in Table 6.

In the simulation of drug interactions, the predicted AUC and C_trough_ showed a 1.68-fold and a 1.94-fold increase in comparison to EM. The ratio of AUC with voriconazole to alone was 5.80. The above data are presented in Figure S7 and Table S7.

### 3.5. Proposal of Initial Dosing Regimen of Tacrolimus

Simulation results showed that as hematocrit increased and weight decreased, oral doses needed to be decreased to maintain optimal exposures (Table 7). For PM, the mean initial daily dose of tacrolimus was 0.08 mg/kg/day. For EM, the mean initial daily dose of tacrolimus was 0.11 mg/kg/day. The initial dose was 20–50% higher in EM than in PM. The recommended mean dose of tacrolimus alone was 0.10 mg/kg/day, and the recommended mean dose of voriconazole was 0.04 mg/kg/day.

## 4. Discussion

This work presented the first PBPK model for heart transplant patients to predict the pharmacokinetics of tacrolimus. In this study, comprehensive clinical data (*n* = 115, Table 2) and external validation data (*n* = 100) were used for the establishment and verification of the model. The data demonstrated that the PK variability of tacrolimus was mainly influenced by fraction unbound in plasma, hematocrit, intrinsic clearance, the proportion of adipose tissue, weight, CYP3A5*3 (rs776746), CYP3A4*18B (rs2242480), IL-10 G-1082A (rs1800896), and concomitant medication. The PBPK model that was established was applied to optimize the dosage regimen of tacrolimus.

The population PK approach (popPK) was used to determine the impact of genetics in tacrolimus pharmacokinetics. In the population pharmacokinetic model, the value of V_d_/F was 656.8 L, which was similar to the data reported in the relevant literature between 532.5 and 846.91 L. CL/F was 12.3 L/h, which is slightly lower than the 14.23–16.87 L/h values that were published in previous studies [20,42], possibly because of the different blood drug concentration detection methods.

It was essential to evaluate the parameters of the PBPK model. The fitted B_max_ was lower than that of liver transplant patients, and there was no difference between K_D_ and liver intrinsic clearance (K_D_ = 3.8 ± 4.7 ng/mL, B_max_ = 418 ± 285 ng/mL, CL_int_ = 10,600 L/h) [26,43]. From Table 3, it can be seen that the absorption rate of heart transplant patients significantly decreased in healthy adults, and the maximum binding capacity of red blood cells and the internal clearance were respectively decreased by 20.8% and 44.3%. Compared to liver transplant patients, heart transplant patients exhibited lower C_max_ and AUC when administered the same dose of tacrolimus, and the impact of hematocrit on the pharmacokinetics of tacrolimus was more significant in heart transplant patients [26,44]. Thus, it may be related to cardiac insufficiency after heart transplantation. The obstruction of systemic circulation will lead to liver congestion and intestinal edema, which will further affect the absorption, distribution, and the quantity and activity of drug-metabolizing enzymes. The CYP3A4 metabolic fraction fm_CYP3A4_ of tacrolimus was 0.35, and the CYP3A5 metabolic fraction fm_CYP3A5_ was 0.55. The CYP3A metabolic fraction fm_CYP3A_ of tacrolimus was 0.9. It was similar to the related research results, which displayed a value of 0.8 [45].

Based on the PBPK model, tacrolimus clearance was influenced by fu_p_, hematocrit, intrinsic clearance, CYP3A5 and CYP3A4 genotypes, and drug–drug interactions; the volume of distribution was determined by bodyweight and the proportion of adipose tissue. In the population analysis of the clinical study, CL/F was related to TBIL, voriconazole, CYP3A5*3 (rs776746), and IL-10 G-1082A (rs1800896); no covariate was found for V_d_/F. The main influencing factors of tacrolimus PK that were determined using the bottom-up approach were different from the results of the population PK analysis. There were several reasons for this discrepancy. Firstly, there is a lack of measurement in clinical studies (fraction unbound in plasma). Additionally, there are limitations concerning detection methods (proportion of adipose tissue, intrinsic clearance). We also have to be aware of that the mean tacrolimus concentration was higher in female patients (3.77 ± 2.82 ng/mL) compared to male patients (2.75 ± 3.31 ng/mL). However, the variability of tacrolimus does not include gender in many studies, which may be due to the large difference in the male to female ratio, with male patients being more commonly administered than female patients [19,20,42,46]. We reduced the number of male patients and performed parameter fitting in the PBPK model after balancing the gender ratio. No statistical differences were found in the fitting results. Previous studies have indicated a higher drug accumulation in female kidney transplant patients [47]. Therefore, it is essential to consider gender as a factor when determining the dose of tacrolimus. These factors were closely associated with weight, the proportion of adipose tissue, and the expression of CYP3A metabolic enzymes. We have incorporated these variables into the PBPK model. Moreover, the limited number of cases in certain studies (weight), and possible confounding effects due to the simultaneous multiple genetic factors (IL-10G-1082A) has demonstrated that IL-10 is a potent modulator of CYP3A enzyme activity, inhibiting CYP3A-associated drug metabolism [48]. In previous studies, tacrolimus metabolism can be affected by the production of IL-10 [49,50]. Specifically, a study on renal transplant patients revealed that the dose of tacrolimus can be adjusted based on the IL-10 G-1082A (rs1800896) genotype [51]. Hence, more research is needed to determine the impact of IL-10 on tacrolimus metabolism in heart transplant patients.

In the recommendation guidelines, the proposed dosing regimen is administration from 0.075 mg/kg/day [4,5]. Our dosage recommendations ranged from 0.075 to 0.110 mg/kg/day in two divided doses (morning and evening). According to CPIC, CYP3A5 expressers (*1*1 or *1*3) require a 2-fold daily dose compared to non-expressers (*3*3) to reach the targeted C_trough_ concentration [10,52]. Regarding CYP3A4, individuals with the CYP3A4*18B variant require a higher tacrolimus dosage than those with the homozygous CYP3A4*1 wild type early after undergoing a transplant [53]. Upon analyzing the genetic information, we found that the CYP3A5*3 mutant was also typically associated with the CYP3A4*18B mutation, indicating a linkage disequilibrium between the two genotypes. The combined effect of CYP3A5*3 and CYP3A4*18B has also been demonstrated [54]. Some studies have suggested that individuals with both CYP3A5*3*3 and CYP3A4*1*1 genotypes require a reduced tacrolimus dose [55]. However, further studies with larger sample sizes are required to confirm the clinical benefits of this.

Pharmacokinetic studies have demonstrated that voriconazole can increase the tacrolimus concentration by inhibiting CYP3A enzyme activity [17]. In DDI simulations, the combination of voriconazole and tacrolimus has a greater impact on the PK of tacrolimus compared to the CYP3A5 genotype (Table S7). The following results have been reported in studies targeting heart transplant patients: the mean dose of tacrolimus after co-administration with voriconazole was 0.02 ± 0.01 mg/kg/day, and the fold changes in concentration and dose were more than 5-fold [39,56]. Currently, there are no definitive guidelines for the combination therapy of tacrolimus and voriconazole. When these two drugs are combined, the dose of tacrolimus should be individualized based on the CYP3A5 genotype of each individual.

The current research suggests that the dosage of tacrolimus should take hematocrit into account [2]. The influence of hematocrit on the tacrolimus concentration was about 29% [20]. In our PBPK model, hematocrit significantly affected the peak concentration, trough concentration, and AUC (Table 5). According to the dose recommendation table (Table 7), it was better to administer 25–40% lower doses to patients with high hematocrit than those with low hematocrit. In clinically unstable transplant patients, fluctuations in red blood cell counts may occur due to factors such as hemorrhage, red blood cell transfusions, and hemolysis, resulting in decreased tacrolimus concentrations that may prompt clinicians to increase the tacrolimus dose. Overall, for clinically stable transplant patients, considering hematocrit levels when normalizing whole blood concentrations of tacrolimus can improve the prediction of whole blood concentrations and help control the free blood concentrations, thus reducing the risk of toxicity [7].

The limitation of this study is that the PBPK model was based on a retrospective data set, and there was also a significant difference in the ratio of men and women, potentially resulting in bias and confounding variables, such as confounding by indication, and surveillance bias. Therefore, prospective studies are necessary to validate these results in future works. Second, the PBPK model did not adequately account for absorption-related physiological factors, such as intestinal permeability. It is necessary to collect more tacrolimus blood concentrations for human intestinal physiology, including tacrolimus absorption and metabolism. In addition, drug interactions between tacrolimus and voriconazole require the collection of blood concentrations and genetic information, such as CYP2C19.

## Figures and Tables

**Table 1:** Physicochemical parameters of tacrolimus used in the PBPK model.

Parameter	Value	Reference
Molecular weight (g/mol)	804.02	Drug label
pK_a_	Neutral	Drug label
Log P	3.3	[26]
fu_p_	0.013	[17]
fm_CYP3A4_	0.35	Calculated
fm_CYP3A5_	0.55	Calculated

Log P: logarithmic of octanol–water partition coefficient; fu_p_: fraction unbound in plasma; fm_CYP3A4_: the fraction of tacrolimus hepatic clearance using CYP3A4; fm_CYP3A5_: the fraction of tacrolimus hepatic clearance using CYP3A5.

**Table 2:** Clinical study: patients’ characteristics.

Characteristics	Median	Range
Age (years)	52.00	46.00, 61.00
Weight (kg)	67.50	57.50, 75.00
BMI (kg/m^2^)	23.30	20.53, 25.36
POD (day)	23.00	19.00, 29.00
Tacrolimus daily dose (mg)	5.00	4.00,6.00
RBC (T/L)	3.46	3.12, 3.93
WBC (T/L)	10.69	9.13, 13.19
HGB (g/L)	103.25	95.26, 115.46
HCT (%)	31.98	29.72, 34.96
ALT (U/L)	27.56	17.37, 39.49
AST (U/L)	19.24	15.92, 23.62
ALP (U/L)	79.18	62.16, 104.62
LDH (U/L)	353.72	294.90, 475.11
CK (U/L)	76.20	50.78, 105.43
ALB (g/L)	36.74	34.48, 38.68
APTT (s)	29.45	28.23, 31.56
PT (s)	13.00	12.51, 13.60
TT (s)	17.55	17.01, 18.14
INR	1.14	1.09, 1.20
TBIL (μmol/L)	15.90	12.13, 21.10
BUN (μmol/L)	10.70	9.19, 15.45
SCR (μmol/L)	86.65	70.94, 128.53
Glucose (μmol/L)	6.61	5.91, 7.96
**Recipient Genotype**	**Count (Percentage)**	***p*-value**
CYP3A5*3 (rs776746)		0.33
CC	38 (44.2%)	
CT	45 (52.3%)	
TT	3 (3.5%)	
CYP3A4*18B (rs2242480)		0.30
CC	45 (52.3%)	
CT	40 (46.5%)	
TT	1 (1.2%)	
IL-10 (rs1800896)		0.20
TT	63 (73.3%)	
CT	23 (26.7%)	

BMI: body mass index; POD: postoperative days; RBC: red blood cell count; WBC: white blood cell count; HGB: hemoglobin; HCT: hematocrit; ALT: alanine transaminase; AST: aspartate aminotransferase; ALP: alkaline phosphatase; LDH: lactate dehydrogenase; CK: phosphocreatine kinase; ALB: albumin concentration; APTT: activated partial thromboplastin time; PT: prothrombin time; TT: thrombin time; INR: international normalized ratio; TBIL: total bilirubin; BUN: blood uric nitrogen; SCR: serum creatinine.

**Table 3:** Final parameter estimates and corresponding residual error based on the PBPK model.

Model Parameter	Healthy Adult Dataset (*n* = 5)		Model Building Dataset (*n* = 115)		External Evaluation Dataset (*n* = 100)	
	Estimate Value	Standard Error	Estimate Value	Standard Error	Estimate Value	Standard Error
K_a_ (1/h)	4.4	0.6	1.9	0.2	1.4	0.1
K_D_ (ng/mL)	6.8	0.5	7.2	0.7	5.9	1.7
B_max_ (ng/mL)	204.8	1.7	145.9	18.4	176.8	45.6
CL_int_ (L/h)	20,706.0	69.7	11,535.0	506.3	10,256.2	314.5

K_a_: first-order absorption rate; K_D_: the affinity constant; B_max_: the binding capacity; CL_int_: the hepatic intrinsic clearance.

**Table 4:** Predicted and observed pharmacokinetic parameters of tacrolimus after oral administration in heart transplant patients.

Dose	AUC_0–12h_			C_max_			Reference
	Mean ± SD Pred (ng × h/mL)	Obs (ng × h/mL)	FE	Mean ± SD Pred (ng/mL)	Obs (ng/mL)	FE	
1.0 mg	50.6 ± 18.7	70.6	0.72	7.3 ± 3.6	10.7	0.68	[41]
2.0 mg	101.3 ± 27.5	116.7	0.87	14.6 ± 4.9	16.3	0.90	[40]
3.0 mg	151.9 ± 33.1	167.2	0.91	21.9 ± 2.5	18.0	1.22	[40]
3.5 mg	177.3 ± 39.6	152.6	1.16	25.6 ± 4.7	24.7	1.04	[40]
4.5 mg	227.9 ± 54.2	230.0	0.99	32.9 ± 5.3	34.7	0.95	[40]

SD: standard deviation; AUC_0–12h_: area under the concentration–time curve from 0 to 12 h; C_max_: peak blood concentration; Pred: predicted value; Obs: observed value; FE: fold error.

**Table 5:** Sensitivity analysis for AUC_0-last_, C_max_, and C_trough_ of tacrolimus.

Input Parameter	Sensitivity Value for AUC_0-last_	Sensitivity Value for C_max_	Sensitivity Value for C_trough_
Fraction unbound in plasma	−1.15	−0.66	−1.66
Hematocrit	0.91	0.67	1.06
Intrinsic clearance (L/h)	−0.74	−0.47	−1.09
Fraction of activity of CYP3A5	−0.30	−0.17	−0.45
Fraction of activity of CYP3A4	−0.15	−0.09	−0.22
Bodyweight (kg)	−0.04	−0.4	−0.13
Proportion of adipose tissue	0.02	0.17	0.05

AUC_0-last_: the area under the curve during a dosing interval; C_max_, peak blood concentration; C_trough_, trough concentration. Sensitivity values less than 0.1 were not listed.

**Table 6:** Predicted pharmacokinetic parameters of tacrolimus after oral administration in heart transplant patients.

Dose Regimen	Population	AUC_0–24_	C_max_	C_trough_
		Pred (ng × h/mL)	Pred (ng/mL)	Pred (ng/mL)
Tacrolimus, 2.5 mg, single dose	EM	119.29 ± 34.00	14.27 ± 3.44	-
	PM	191.91 ± 40.74	20.32 ± 3.55	-
Tacrolimus, 2.5 mg, q12h	EM	-	23.08 ± 3.86	9.62 ± 2.47
	PM	-	40.37 ± 5.13	21.00 ± 3.24

Data are shown as the mean ± SD; AUC_0–24_: area under the concentration–time curve from 0 to 24 h; C_max_: peak blood concentration; C_trough_: steady-state trough concentration; Pred: predicted value; EM: extensive metabolizers (CYP3A5*1*1 or *1*3 and CYP3A4*18B*18B or CYP3A4*1*18B); PM: poor metabolizers (CYP3A5*3*3 and CYP3A4*1*1).

**Table 7:** Dosing regimens (q12h, mg) for heart transplant recipients with CYP3A5 and CYP3A4 genotypes based on BW, hematocrit, and drug combination.

**BW < 50 kg**	**PM**		**EM**	
Drug combination	Hematocrit	Hematocrit	Hematocrit	Hematocrit
	(0.2–0.3)	(0.3–0.4)	(0.2–0.3)	(0.3–0.4)
Without voriconazole	2.5 (92.5) *	1.8 (90.7) *	4.0 (94.8) *	2.8 (96.1) *
With voriconazole	1.0 (91.8) *	0.5 (92.4) *	2.0 (90.7) *	1.0 (94.5) *
**BW = 50–80 kg**	**PM**		**EM**	
Drug combination	Hematocrit (0.2–0.3)	Hematocrit (0.3–0.4)	Hematocrit (0.2–0.3)	Hematocrit (0.3–0.4)
Without voriconazole	3.0 (93.8) *	2.5 (96.3) *	4.5 (93.3) *	3.0 (95.7) *
With voriconazole	1.5 (92.9) *	1.0 (94.1) *	2.5 (90.5) *	1.5 (91.9) *
**BW > 80 kg**	**PM**		**EM**	
Drug combination	Hematocrit	Hematocrit	Hematocrit	Hematocrit
	(0.2–0.3)	(0.3–0.4)	(0.2–0.3)	(0.3–0.4)
Without voriconazole	3.5 (93.2) *	2.5 (91.8) *	5.0 (95.3) *	3.2 (94.2) *
With voriconazole	1.5 (92.5) *	1.0 (94.4) *	2.5 (93.7) *	2.0 (94.0) *

* Percentage of trough concentrations within target concentrations during therapy; BW: bodyweight; EM: extensive metabolizers (CYP3A5*1*1 or *1*3 and CYP3A4*18B*18B or CYP3A4*1*18B); PM: poor metabolizers (CYP3A5*3*3 and CYP3A4*1*1).

## Data Availability

The data presented in this study are available upon request from the corresponding authors. The data are not publicly available due to privacy or ethical restrictions.

## Supplementary Materials

The following supporting information can be downloaded at: https://www.mdpi.com/article/10.3390/pharmaceutics15112580/s1, Table S1: Human Characteristics and physicochemical Parameters values in the tacrolimus PBPK model. Table S2: Predicted tacrolimus tissue-to-plasma partition coefficients using the Rodgers et al.’s method. Table S3: Results of genotyping and Hardy-Weinberg equilibrium analysis (*n* = 86). Table S4: Final popPK model characteristics and bootstrap results. Table S5: Predicted and observed pharmacokinetic parameters of tacrolimus after oral administration in healthy adults. Table S6: Drug constants and physiological parameter values of Voriconazole used in the PBPK model. Table S7: Predicted pharmacokinetic parameters of tacrolimus after oral administration in heart transplant patients. Figure S1: Flow diagram used for initial detailed PBPK model of tacrolimus. Figure S2: Prediction-corrected visual predictive check (pcVPC) obtained from 1000 simulations of the database. Figure S3: Linear analysis for healthy adults of the predicted concentration and observed concentration of tacrolimus. Figure S4: Simulation of concentration-time profiles of voriconazole after a single oral dose of 400 mg in Chinese healthy adults. Figure S5: Simulation of concentration-time profiles of tacrolimus in combination with voriconazole in heart transplant recipients. Figure S6: Linear analysis for healthy adults and heart transplant recipients of the predicted concentration and observed concentration of tacrolimus in combination with voriconazole. Figure S7: Predicted blood concentration-time profiles of tacrolimus after a single oral dose of 2.5 mg tacrolimus combined with 200 mg voriconazole (A); predicted blood concentration-time profiles of tacrolimus after multidose of tacrolimus (2.5 mg, q12h for 6 days) combined with voriconazole (200 mg, q12h, day 4–6) (B). TAC, tacrolimus; EM: extensive metabolizers (CYP3A5*1*1 or *1*3 and CYP3A4*18B*18B or CYP3A4*1*18B); PM: poor metabolizers (CYP3A5*3*3 and CYP3A4*1*1). References [17,24,25,28,29,57] are cited in Supplementary Materials.
