# Supplementary material for: A Physiologically Based Pharmacokinetic Approach to Recommend an Individual Dose of Tacrolimus in Adult Heart Transplant Recipients
Source: Pharmaceutics. 2023 Nov 3;15(11):2580. doi: 10.3390/pharmaceutics15112580 (PMC10675244; doi:10.3390/pharmaceutics15112580)
Supplement: Supplementary file 1 [file pharmaceutics-15-02580-s001.zip › Supplementary material/Supplementary material.pdf]

SUPPLEMENTARY MATERIAL: A Physiologically-Based Pharmacokinetic Approach to Recommend Individual Dose of Tacrolimus in Adult Heart Transplant Recipients

Ling Pei, Run Li, Hong Zhou, Wenxin Du, Yajie Gu Yingshuo Jiang, Yongqing Wang, Xin Chen, Jianguo Sun, Junrong Zhu

1. Model Development

1.1 The schematic diagram of the PBPK model.
1.2 Human anatomical and physiological parameters.
1.3 The tissue-to-plasma partition coefficients.

2. Population PK Study

2.1 Population PK model.
2.2 The genetic information.
2.3 The parameter estimates of the final model and bootstrap median estimates.
2.4 Prediction-corrected visual predictive check (pcVPC).

3. Model Evaluation

3.1 Linear analysis for healthy adults of the predicted concentration and observed concentration of tacrolimus.
3.2 The pharmacokinetics (AUC, C_{max}) in healthy adults.

4. Drug-Drug Interaction (DDI) Model

4.1 DDI Model Development and Evaluation
4.2 DDI Model Simulation.

1. Model Development

1.1 The schematic diagram of the PBPK model.

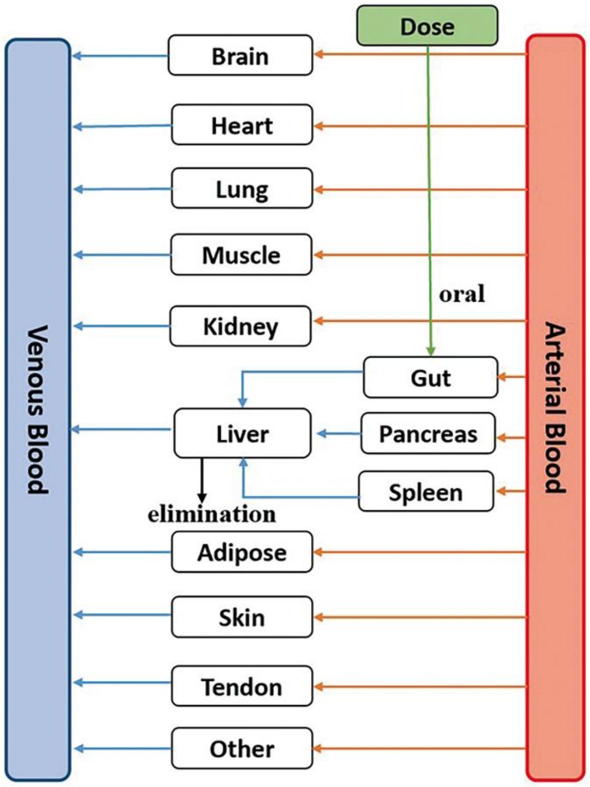

Figure S1. Flow diagram used for initial detailed PBPK model of tacrolimus.

1.2 Human anatomical and physiological parameters.

Table S1. Human Characteristics and physicochemical Parameters values in the tacrolimus PBPK model.

Organ	Weight (kg)	Blood Flow (L/h/kg)	Reference
Artery	1.1	/	[1, 2]
Vein	4.4	/	[1, 2]
Gut	1.17	50	[1, 2]
Spleen	0.15	78	[1, 2]
Pancreas	0.14	27.9	[1, 2]
Liver	1.8	15	[1, 2]
Muscle	26	1.4	[1, 2]
Kidney	0.31	240	[1, 2]
Brain	1.4	33.6	[1, 2]
Heart	0.33	48	[1, 2]
Lung	0.54	629	[1, 2]
Skin	2.6	6	[1, 2]
Tendon	3	0.6	[1, 2]
Other	5.56	1.2	[1, 2]
Adipose	17.5	2.6	[1, 2]
Bone	4	0	[1, 2]

1.3 The tissue-to-plasma partition coefficients.

Table S2. Predicted tacrolimus tissue-to-plasma partition coefficients using the Rodgers et al.'s method.

Parameter	Value	Reference
Adipose	1.9	[3, 4]
Bone	1.89	[3, 4]
Brain	1.91	[3, 4]
Gut	1.56	[3, 4]
Heart	0.74	[3, 4]
Kidney	0.96	[3, 4]
Liver	1.33	[3, 4]
Lung	0.52	[3, 4]
Pancreas	1.37	[3, 4]
Muscle	0.96	[3, 4]
Skin	1.07	[3, 4]
Spleen	0.97	[3, 4]
Additional Organ	1.9	[3, 4]

2. Population PK Study

2.1 Population PK model

2.1.1. Base Model

Population PK analysis was conducted with the Phoenix NLME 8.3 program (Certara, St Louis, Missouri, USA). The first-order conditional estimation (FOCE) algorithm was applied throughout the modeling process. A one-compartment model with first-order absorption and elimination was evaluated to fit the tacrolimus concentration. The best structural model was chosen based on visual inspection of the data and the values of the objective function value (OFV), Akaike Information Criterion (AIC), and Bayesian Information Criterion (BIC). The interindividual variability was described by an exponential model (Eq. S1).

$$P_i = P_t \times \exp(\eta_i) \quad \text{(S1)}$$

In this equation, where P_i represents the individual parameter estimate, P_t represents the population typical value of this parameter, η_i is a random variable normally distributed with a mean of zero and variances of ω^2.

The proportional model was suitable for the description of intraindividual variability (residual error).

$$C_i = C \times (1+\varepsilon) \quad \text{(S2)}$$

The observations (C_i) and predictions (C) were analyzed using the proportional model for the description of residual error (ε). The error was assumed to be normally distributed with mean zero and variances of σ^2. Categorical covariates, such as SEX (male=1 and female=2) and Voriconazole (co-administration=1 and none=0), were included in the analysis using indicator variables.

2.1.2. Covariate Model

The covariate model was constructed to determine covariates using a stepwise forward inclusion ($p<0.05$) backward exclusion ($p<0.01$) regression approach. It was evaluated using prediction corrected visual predictive check (pcVPC) and the bootstrap approach. When two or more covariates were found to significantly improve the model, the covariate causing the largest reduction was left in the final model.

2.2 The genetic information.

Table S3. Results of genotyping and Hardy-Weinberg equilibrium analysis (n=86).

	SNP	genotype	N (%)	P-value
SLCO1B1	rs2306283	AG	42 (49%)	0.32
		GG	42 (49%)	
		AA	2 (2%)	
SLCO1B1	rs2291075	TT	17 (20%)	0.37
		CT	51 (59%)	
		CC	18 (21%)	
IL-10	rs1800871	AA	37 (43%)	0.34
		AG	44 (51%)	
		GG	5 (6%)	
IL-10	rs1800872	GG	6 (7%)	0.34
		TT	37 (43%)	
		TG	43 (50%)	
IL-6	rs1800796	GC	48 (56%)	0.36
		CC	30 (35%)	
		GG	8 (9%)	
CYP2J2	rs890293	CC	75 (87%)	0.11
		CA	11 (13%)	
ABCB1	rs1128503	AG	55 (64%)	0.37
		AA	23 (27%)	
		GG	8 (9%)	
ABCB1	rs2032582	AA	10 (12%)	0.69 (A/T)

		CC	17 (20%)	0.53 (A/C)
		TT	1 (1%)	0.36 (C/T)
		AC	31 (36%)	
		AT	15 (17%)	
		CT	12 (14%)	
ABCB1	rs1045642	GG	30 (35%)	0.35
		AG	51 (59%)	
		AA	5 (6%)	
CYP3A7	rs2257401	GC	48 (56%)	0.34
		GG	35 (41%)	
		CC	3 (3%)	
POR*28	rs1057868	CC	35 (41%)	0.34
		CT	49 (57%)	
		TT	2 (2%)	
PPARα	rs4823613	AA	49 (57%)	0.3
		GG	4 (5%)	
		AG	33 (38%)	

Note: A results of genotyping and Hardy Weinberg equilibrium analysis; The rs2032582 locus has multiple types (AA, CA, CC, CT, TT, AT) and is divided into three groups: A/C, A/T, and C/T for analysis. SNP, single nucleotide polymorphisms.

2.3 The parameter estimates of the final popPK model and bootstrap median estimates.

Table S4. Final popPK model characteristics and bootstrap results.

Population pharmacokinetic parameters	Final model		Bootstrap (n = 1000)	
	Estimate	CV%	Median	95% CI
K_a(1/h)	0.30(fixed)	-	0.30(fixed)	-
V_d/F(L)	656.80	13.65	669.13	511.39-744.68
CL/F (L/h)	12.35	7.17	12.00	11.15-13.00
TBIL	-0.19	-18.78	-0.19	-0.26 to -0.14
Voriconazole	-0.64	-32.19	-0.64	-0.91 to -0.44
rs776746-TT	1.18	13.94	1.17	0.99-1.62
rs776746-TC	0.77	11.59	0.79	0.69-0.86
rs1800896-TC	-0.35	-27.58	-0.34	-0.44 to -0.19
Interindividual variability				
$\omega_{Vd/F}^2$	0.70	35.7	0.80	0.23-1.36
$\omega_{CL/F}^2$	0.14	14.3	0.13	0.09-0.17
Residual variability				
Proportional (%)	1.48	5.7	1.49	1.36-1.58

K_a: first-order absorption rate (1/h); V_d/F, apparent volume of distribution (L); CL/F, apparent clearance rate (L/h); TBIL, total bilirubin; CV, coefficient of variation; CI, confidence interval.

2.4 Prediction-corrected visual predictive check (pcVPC).

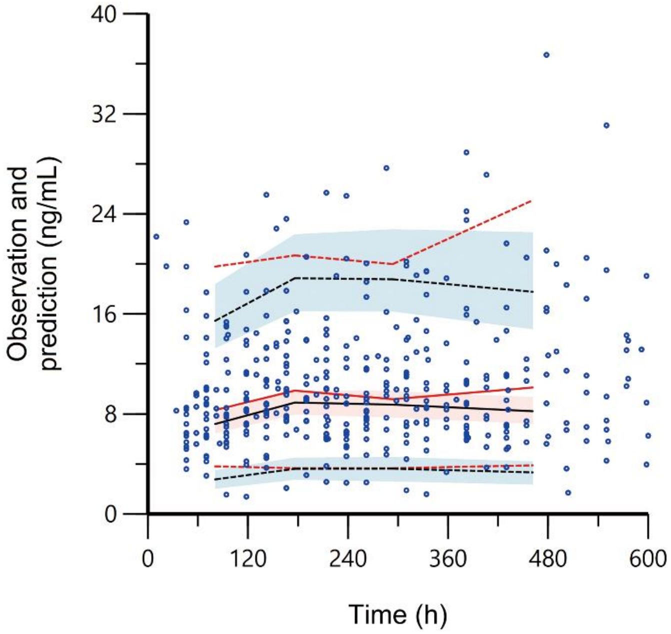

Figure S2. Prediction-corrected visual predictive check (pcVPC) obtained from 1000 simulations of the database. The dots are the prediction-corrected drug concentrations. The black solid line represents predicted 50th percentile and the black dotted lines mean the 5th and 95th percentiles. Shadow means the areas between the 5th and 95th percentiles representing the 90% prediction interval. The red solid line means the observed 50thpercentile and the red dashed lines are the 5th and 95th percentiles of observations.

3. Model Evaluation

3.1 Linear analysis for healthy adults of the predicted concentration and observed concentration of tacrolimus.

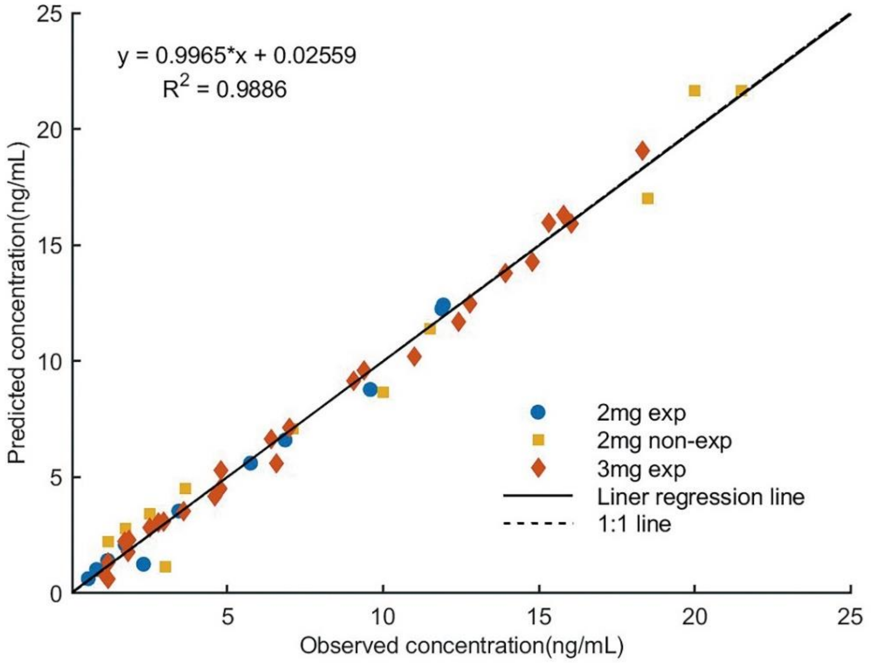

Figure S3. Linear analysis for healthy adults of the predicted concentration and observed concentration of tacrolimus.

3.2 The pharmacokinetics (AUC, C_{max}) in healthy adults.

Table S5. Predicted and observed pharmacokinetic parameters of tacrolimus after oral administration in healthy adults.

Dose	Population	AUC_{0-24h}			C_{max}			Reference
		Mean±SD Pred (ng*h/mL)	Obs (ng*h/mL)	FE	Mean±SD Pred (ng/mL)	Obs (ng/mL)	FE	
2.0mg	Exp	59.6±20.9	60.8	0.98	12.4±3.5	12.1	1.02	[5]
	Non-exp	110.4±42.8	119.1	0.93	21.1±3.2	24.7	0.85	[5]
3.0mg	Exp	90.2±23.7	91.6	0.98	17.8±8.1	17.3	1.03	[6]
	Non-exp	180.3±48.4	-	-	26.7±9.6	-	-	-

SD: standard deviation; AUC_{0-24h}: area under the concentration time curve from 0 to 24 hours; C_{max}: peak blood concentration; Exp: CYP3A5 expressers; Non-exp: CYP3A5 non-expressers; Pred: predicted value; Obs: observed value; FE: Fold error.

4. Drug-Drug Interaction (DDI) Model

4.1 DDI Model Development and Evaluation

Voriconazole was identified to cause reversible inhibition and time-dependent inhibition of CYP3A enzyme [7, 8]. To simplify the model, we used the reversible inhibition model. Therefore, reversible inhibition (Eq. 1) was tested in this study. Moreover, tacrolimus clearance of drug in-teractions was tested (Eq. 2).

$$DDI_{RE} = \frac{1}{1+\frac{C_{b,vor}\times fu_{b,vor}}{KI}} \quad (1)$$

$$CL_{liver} = [(fm_{CYP3A5}\times FA_{CYP3A5}+fm_{CYP3A4}\times FA_{CYP3A4})\times DDI_{RE}+fm_{other}] \times Q_{liver}\times E \quad (2)$$

where DDI = the inhibition ratio of voriconazole on CYP3A, RE = the reversible inhibition, $C_{b,vor}$ = whole blood concentration of voriconazole; $fu_{b,vor}$ = unbound fraction in blood of voriconazole; KI = the inhibition constant of voriconazole for CYP3A. The parameters of voriconazole in the model were listed in Table S6.

Table S6. Drug constants and physiological parameter values of Voriconazole used in the PBPK model.

Parameter	Value	Reference
Molecular weight (g/mol)	349.31	Drug label
pK_a	1.60 (base)	[7]
Log P	1.80	[7]
fu_p	0.42	[7]
BPR	1.00	[8]
KI (ng/mL)	8.70	Predicted
CL_{int} (L/h)	273.21	Predicted

Log P: logarithmic of octanol-water partition coefficient; fu_p: fraction unbound in plasma; KI: the inhibition constant of voriconazole for CYP3A inhibition; CL_{int}: intrinsic clearance.

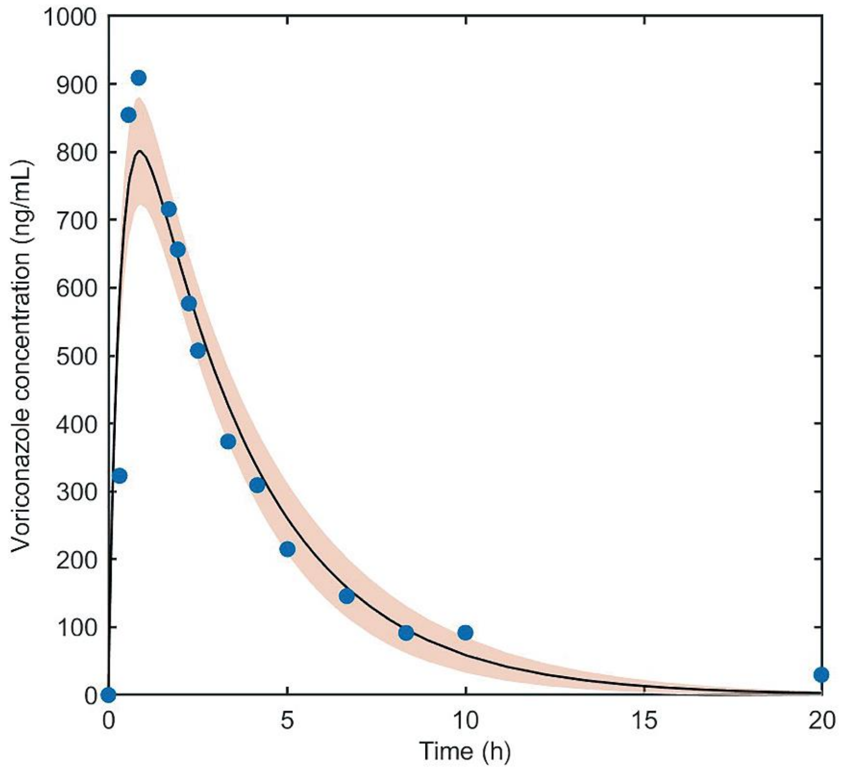

Figure S4. Simulation of concentration-time profiles of voriconazole after a single oral dose of 400 mg in Chinese healthy adults. Solid line and the shaded section represent the mean and 5th–95th percentiles of simulated population, respectively. Blue circles represent the means of observed data.

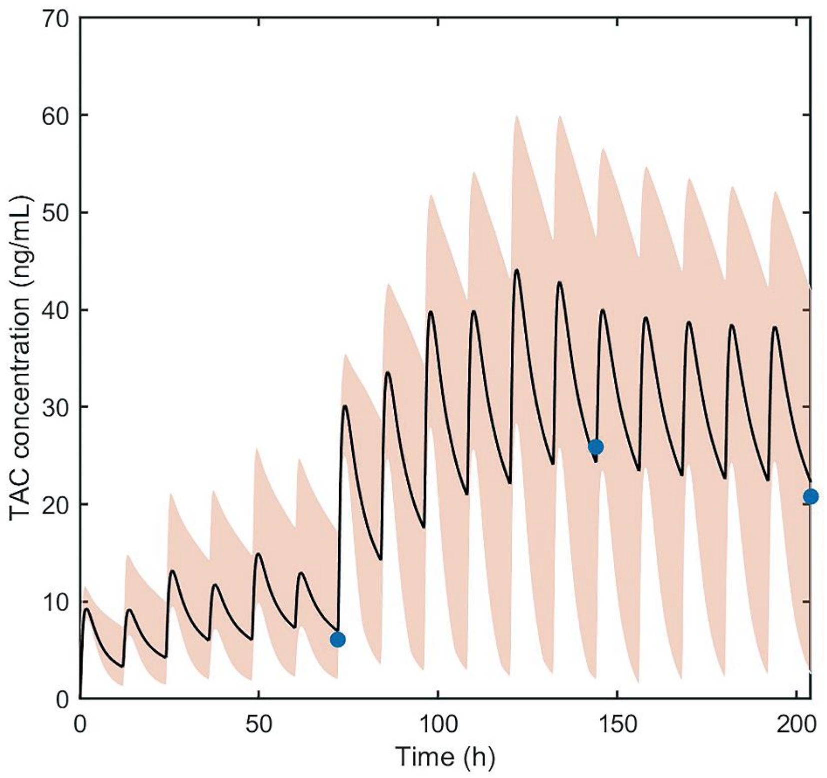

Figure S5. Simulation of concentration-time profiles of tacrolimus in combination with voriconazole in heart transplant recipients. Solid line and the shaded section represent the mean and 5th–95th percentiles of simulated population, respectively. Blue circles represent the means of observed data.

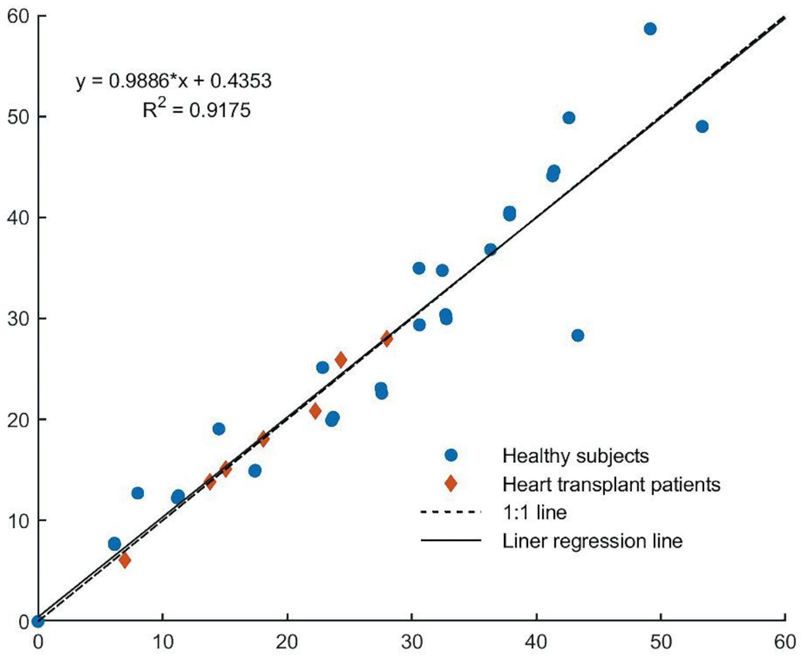

Figure S6. Linear analysis for healthy adults and heart transplant recipients of the predicted concentration and observed concentration of tacrolimus in combination with voriconazole.

4.2 DDI Model Simulation.

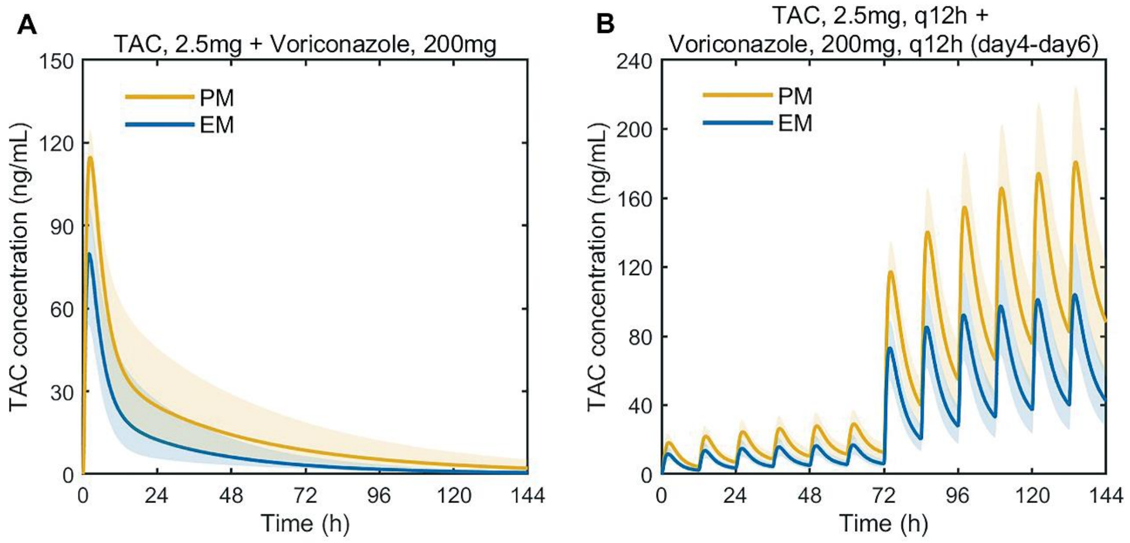

Figure S7. Predicted blood concentration-time profiles of tacrolimus after a single oral dose of 2.5 mg tacrolimus combined with 200 mg voriconazole (**A**); predicted blood concentration-time profiles of tacrolimus after multidose of tacrolimus (2.5 mg, q12h for 6 days) combined with voriconazole (200 mg, q12h, day 4-6) (**B**). TAC, tacrolimus; EM: extensive metabolizers (CYP3A5 *1*1 or *1*3 and CYP3A4 *18B*18B or CYP3A4 *1*18B); PM: poor metabolizers (CYP3A5 *3*3 and CYP3A4 *1*1). The thick line represents the mean predicted data; the shadow represents the 5th and 95th percentiles of the prediction.

Table S7. Predicted pharmacokinetic parameters of tacrolimus after oral administration in heart transplant patients.

Dose Regimen	Population	AUC_{0-24}	C_{max}	C_{trough}
		Pred (ng*h/mL)	Pred (ng/mL)	Pred (ng/mL)
Tacrolimus, 2.5mg +	EM	676.16±289.36	79.83±4.18	-
Voriconazole, 200mg	PM	1133.00±574.20	114.72±4.86	-
Tacrolimus, 2.5mg, q12h +	EM	-	133.92±2.57	46.14±3.16
Voriconazole, 200 mg, q12h, day 4-6	PM	-	180.91±2.94	89.76±4.72

Data are shown as the mean±SD; AUC_{0-24}: area under the concentration time curve from 0-24 hours; C_{max}: peak blood concentration; C_{trough}: steady-state trough concentration; Pred: predicted value; EM: extensive metabolizers (CYP3A5 *1*1 or *1*3 and CYP3A4 *18B*18B or CYP3A4 *1*18B); PM: poor metabolizers (CYP3A5 *3*3 and CYP3A4 *1*1).

References

1 Levitt, D. G. PKQuest: A General Physiologically Based Pharmacokinetic Model. Introduction and Application to Propranolol. *BMC Clin Pharmacol* **2002**, *2*, 5. https://doi.org/10/dghx49.

2 Levitt, D. G.; Schnider, T. W. Human Physiologically Based Pharmacokinetic Model for Propofol. *BMC Anesthesiol* **2005**, *5*, 4. https://doi.org/10/bcszrz.

3 El-Khateeb, E.; Chinnadurai, R.; Al Qassabi, J.; Scotcher, D.; Darwich, A. S.; Kalra, P. A.; Rostami-Hodjegan, A. Using Prior Knowledge on Systems Through PBPK to Gain Further Insight into Routine Clinical Data on Trough Concentrations: The Case of Tacrolimus in Chronic Kidney Disease. *Therapeutic Drug Monitoring* **2023**, *Publish Ahead of Print*. https://doi.org/10.1097/FTD.0000000000001108.

4 Rodgers, T.; Rowland, M. Physiologically Based Pharmacokinetic Modelling 2: Predicting the Tissue Distribution of Acids, Very Weak Bases, Neutrals and Zwitterions. *J Pharm Sci* **2006**, *95*, 1238–1257. https://doi.org/10.1002/jps.20502.

5 He, Q.; Bu, F.; Zhang, H.; Wang, Q.; Tang, Z.; Yuan, J.; Lin, H.-S.; Xiang, X. Investigation of the Impact of CYP3A5 Polymorphism on Drug-Drug Interaction between Tacrolimus and Schisantherin A/Schisandrin A Based on Physiologically-Based Pharmacokinetic Modeling. *Pharmaceuticals (Basel)* **2021**, *14*, 198. https://doi.org/10.3390/ph14030198.

6 Imamura, C. K.; Furihata, K.; Okamoto, S.; Tanigawara, Y. Impact of Cytochrome P450 2C19 Polymorphisms on the Pharmacokinetics of Tacrolimus When Coadministered with Voriconazole. *J Clin Pharmacol* **2016**, *56*, 408–413. https://doi.org/10.1002/jcph.605.

7 Dong, J.; Liu, S.; Rasheduzzaman, J. M.; Huang, C.; Miao, L. Development of Physiology Based Pharmacokinetic Model to Predict the Drug Interactions of Voriconazole and Venetoclax. *Pharm Res* **2022**, *39*, 1921–1933. https://doi.org/10.1007/s11095-022-03289-9.

8 Li, Z.; Shen, C.; Li, R.; Wang, B.; Li, J.; Niu, W.; Zhang, L.; Zhong, M.; Wang, Z.; Qiu, X. Individual Dose Recommendations for Drug Interaction Between Tacrolimus and Voriconazole in Adult Liver Transplant Recipients. *European Journal of Pharmaceutical Sciences* **2023**, 106405. https://doi.org/10.1016/j.ejps.2023.106405.
