# Supplementary figures and images for: A Physiologically Based Pharmacokinetic Approach to Recommend an Individual Dose of Tacrolimus in Adult Heart Transplant Recipients
Source: Pharmaceutics. 2023 Nov 3;15(11):2580. doi: 10.3390/pharmaceutics15112580 (PMC10675244; doi:10.3390/pharmaceutics15112580)

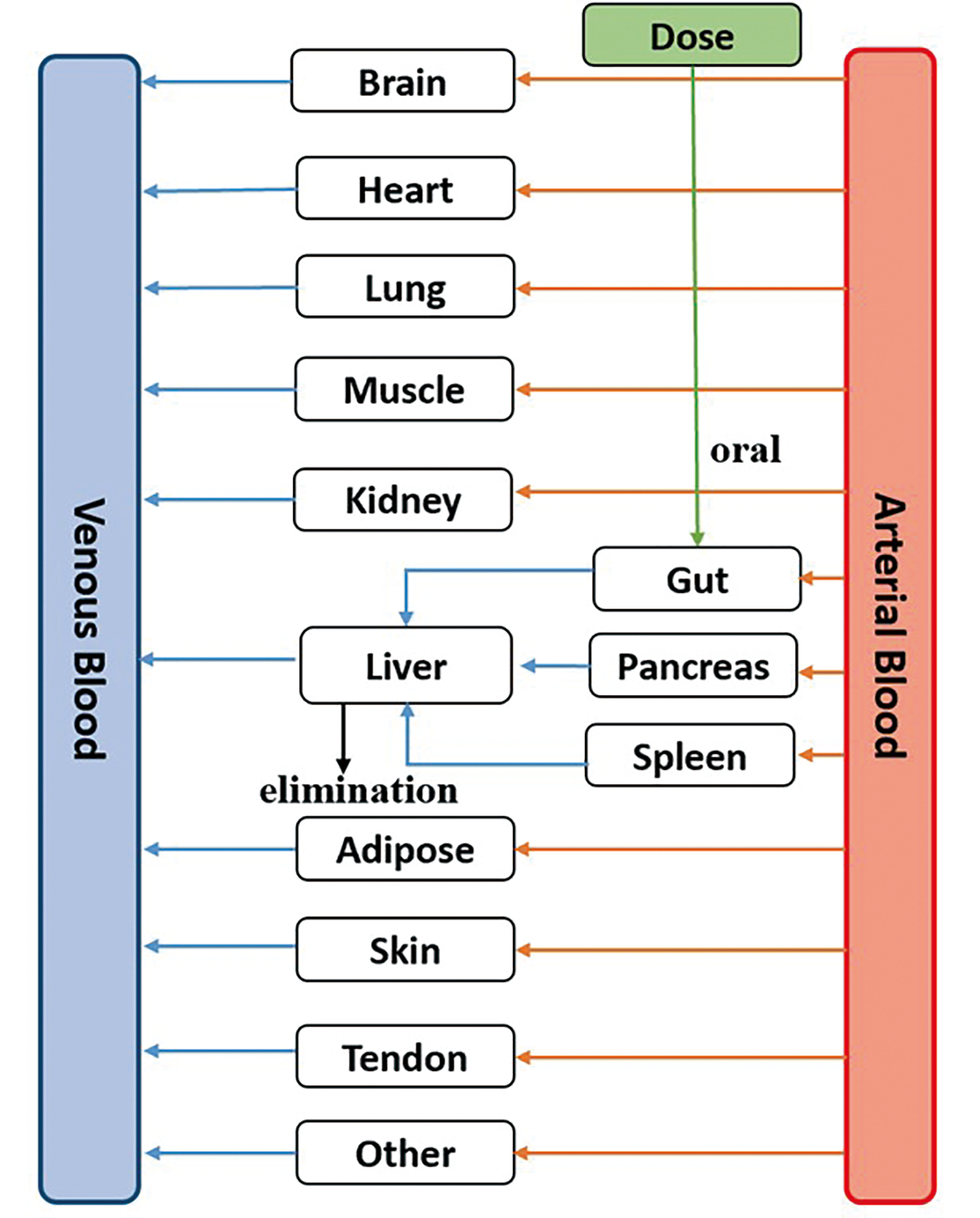

Supplement: Supplementary file 1 [file pharmaceutics-15-02580-s001.zip › Supplementary material/Figure S1.png]

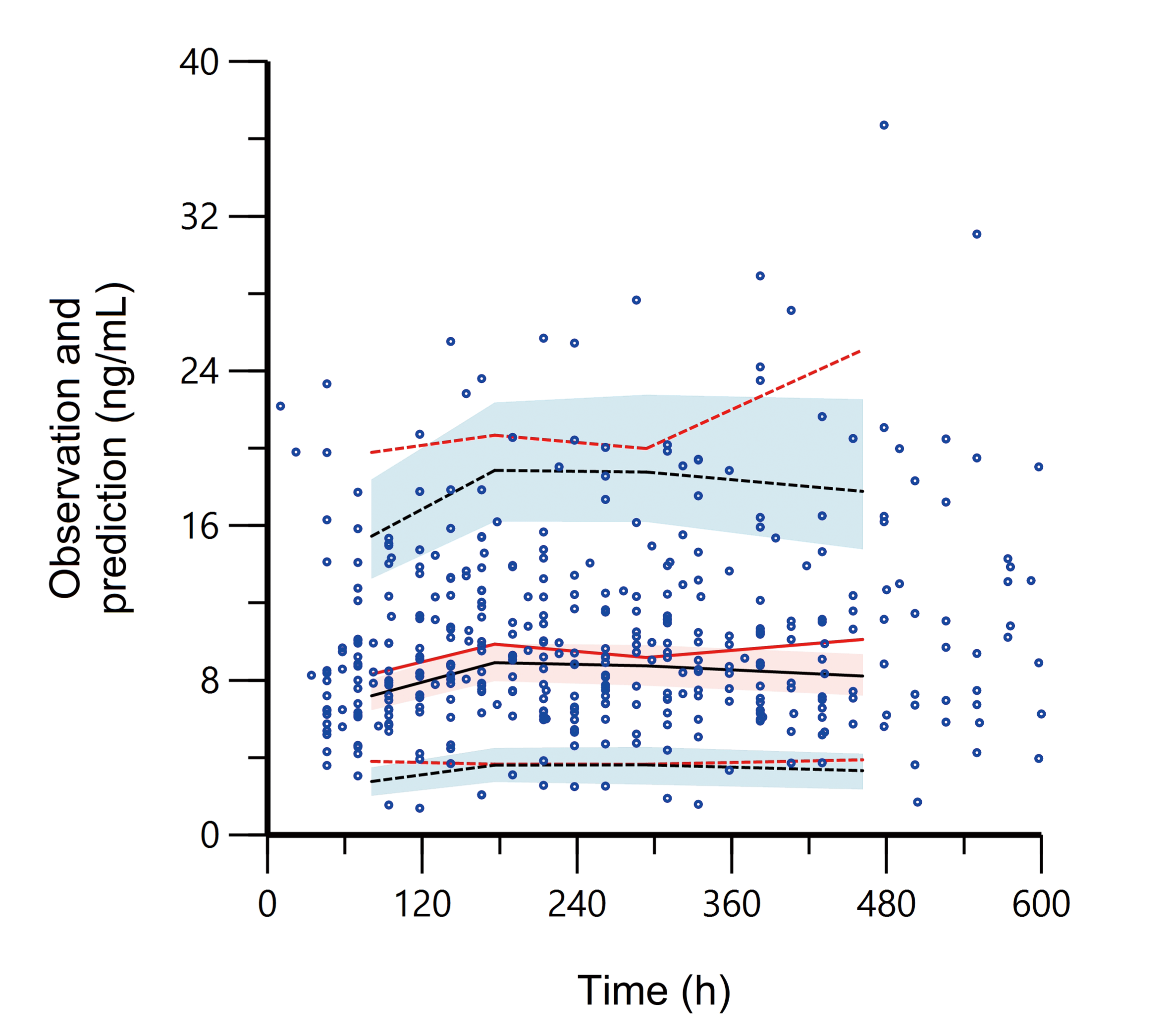

Supplement: Supplementary file 1 [file pharmaceutics-15-02580-s001.zip › Supplementary material/Figure S2.png]

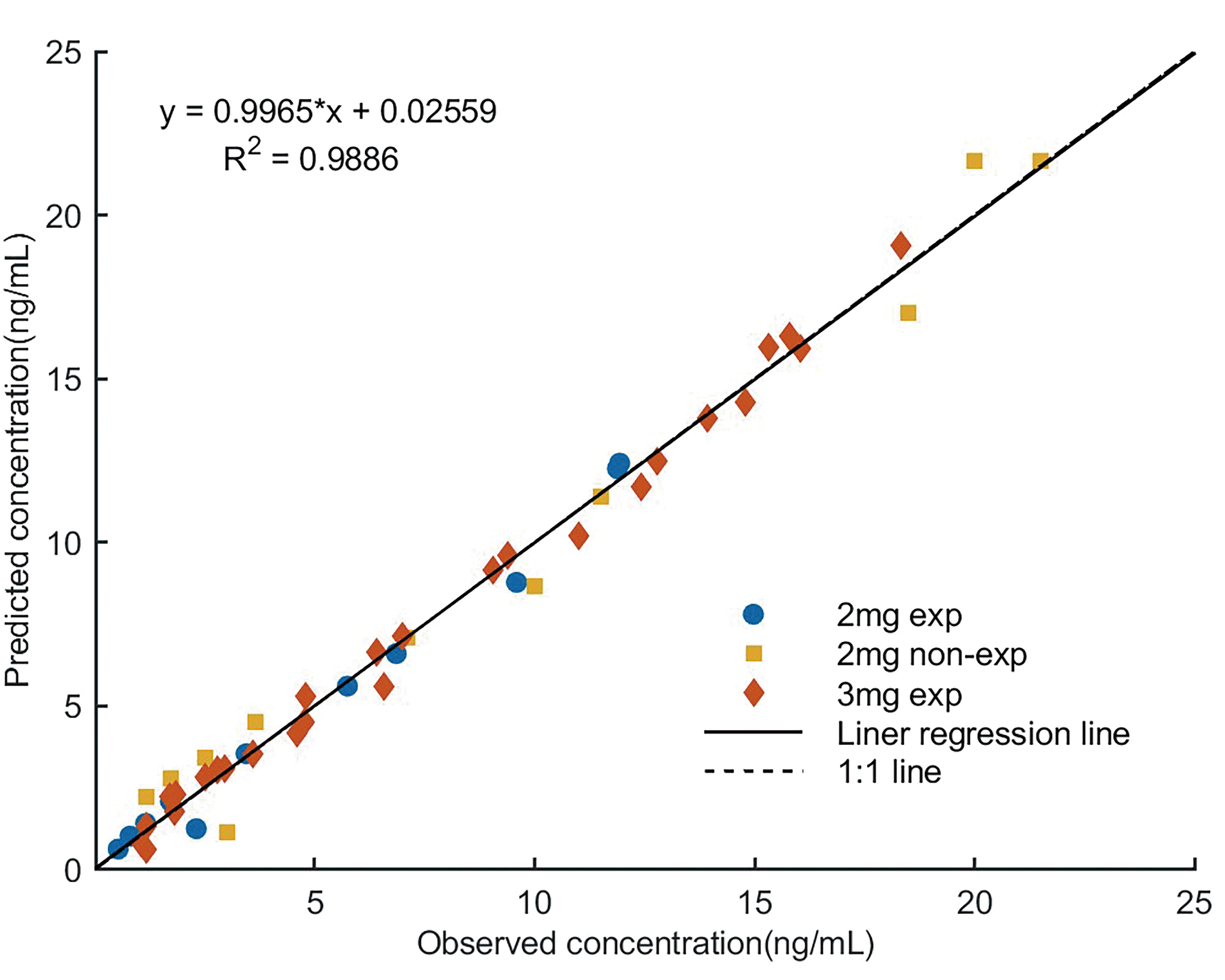

Supplement: Supplementary file 1 [file pharmaceutics-15-02580-s001.zip › Supplementary material/Figure S3.png]

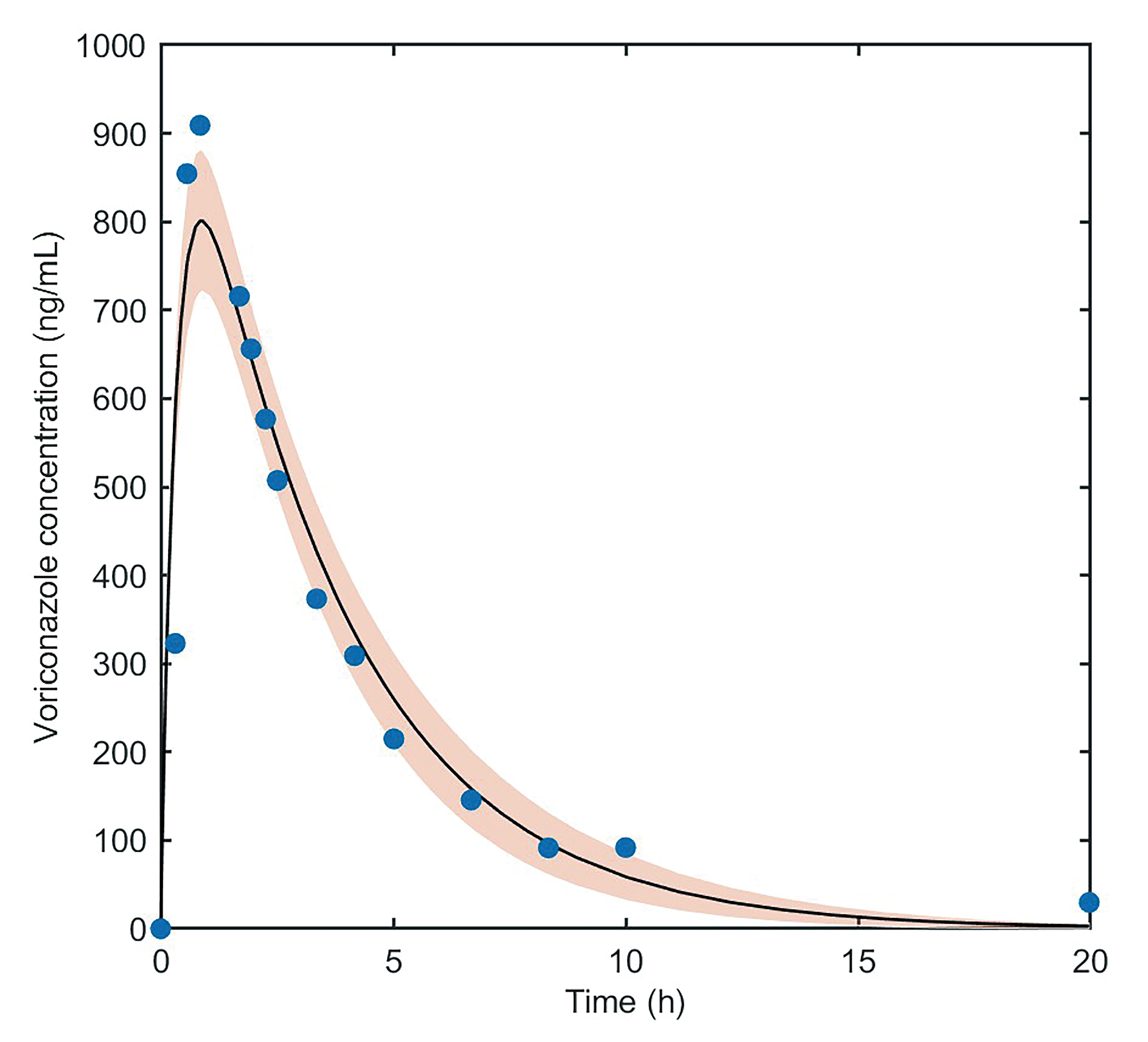

Supplement: Supplementary file 1 [file pharmaceutics-15-02580-s001.zip › Supplementary material/Figure S4.png]

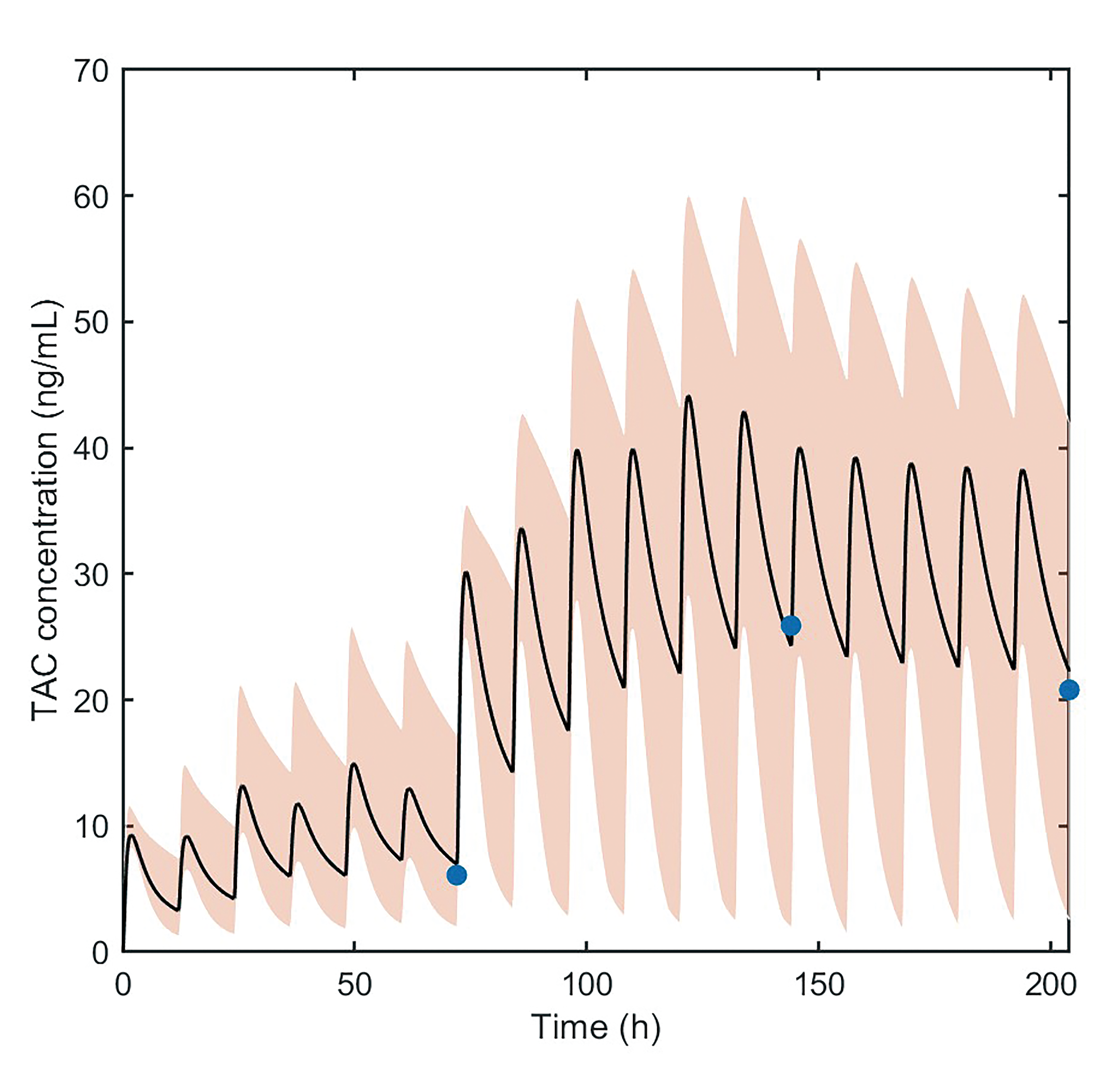

Supplement: Supplementary file 1 [file pharmaceutics-15-02580-s001.zip › Supplementary material/Figure S5.png]

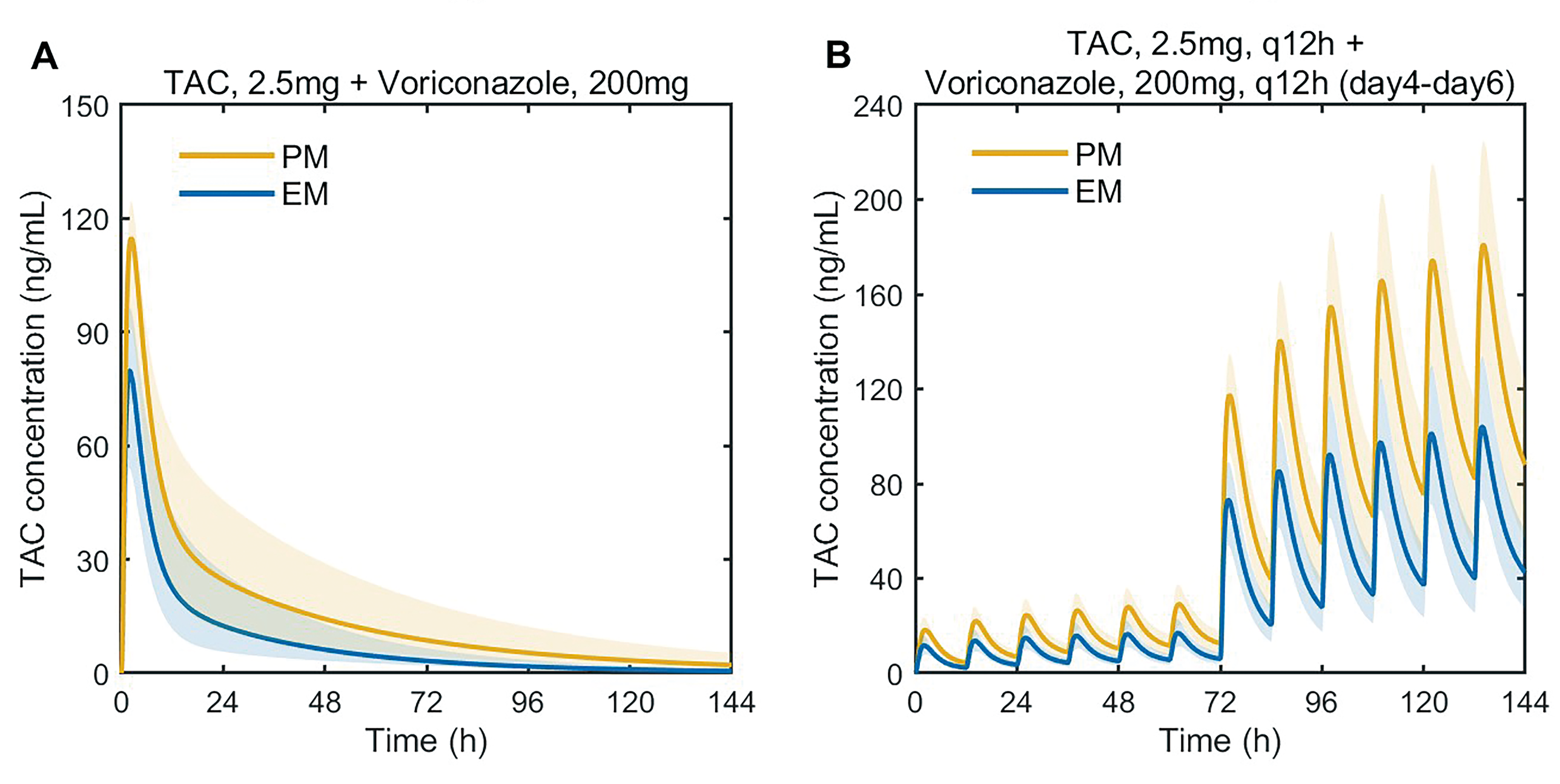

Supplement: Supplementary file 1 [file pharmaceutics-15-02580-s001.zip › Supplementary material/Figure S7.png]
